# Genetic analysis of medaka fish illuminates conserved and divergent roles of Pax6 in vertebrate eye development

**DOI:** 10.3389/fcell.2024.1448773

**Published:** 2024-10-24

**Authors:** Simona Mikula Mrstakova, Zbynek Kozmik

**Affiliations:** Laboratory of Transcriptional Regulation, Institute of Molecular Genetics of the Czech Academy of Sciences, Prague, Czechia

**Keywords:** lens, retina, eye evolution, vision, gene expression, Pax6

## Abstract

Landmark discovery of eye defects caused by Pax6 gene mutations in humans, rodents, and even fruit flies combined with Pax6 gene expression studies in various phyla, led to the master control gene hypothesis postulating that the gene is required almost universally for animal visual system development. However, this assumption has not been broadly tested in genetically trackable organisms such as vertebrates. Here, to determine the functional role of the fish orthologue of mammalian Pax6 in eye development we analyzed mutants in medaka Pax6.1 gene generated by genome editing. We found that transcription factors implicated in vertebrate lens development (Prox1a, MafB, c-Maf, FoxE3) failed to initiate expression in the presumptive lens tissue of Pax6.1 mutant fish resulting in aphakia, a phenotype observed previously in Pax6 mutant mice. Surprisingly, the overall differentiation potential of Pax6.1-deficient retinal progenitor cells (RPCs) is not severely compromised, and the only cell types affected by the absence of Pax6.1 transcription factor are retinal ganglion cells. This is in stark contrast to the situation in mice where the Pax6 gene is required cell-autonomously for the expansion of RPCs, and the differentiation of all retina cell types. Our results provide novel insight into the conserved and divergent roles of Pax6 gene orthologues in vertebrate eye development indicating that the lens-specific role is more evolutionarily conserved than the role in retina differentiation.

## Introduction

Landmark discovery of *Pax6* gene mutations in humans, rodents and fruit fly *Drosophila melanogaster* (Hill et al., 1991; Ton et al., 1991; Glaser et al., 1992; Quiring et al., 1994; Czerny et al., 1999), which all lead to defects in eye development, challenged the contemporary view that widely different anatomical designs arose independently during evolution. Since then, more evidence, mostly based on gene expression studies, has emerged in favour of the redeployment of *Pax6* genes within the genetic program underlying eye formation throughout the animal kingdom (Kozmik, 2005; Cvekl and Callaerts, 2017). In model organims allowing genetic approaches such as the laboratory mouse or fruit fly we begin to understand function of *Pax6* during animal eye development at mechanistic level, by defining its role in cell proliferation, in cell type differentiation, and in participation in complex gene regulatory networks (Shaham et al., 2012; Cvekl and Callaerts, 2017). Phenotypic studies of *Pax6* mutants performed in selected vertebrate species, such as zebrafish (Kleinjan et al., 2008; Takamiya et al., 2015; Takamiya et al., 2020), mouse (Hill et al., 1991; Ashery-Padan et al., 2000; Marquardt et al., 2001; Klimova and Kozmik, 2014), rat (Matsuo et al., 1993) or *Xenopus* (Nakayama et al., 2015) provided key insight into the role of *Pax6* in visual system development. The vertebrate eye is predominantly composed of the derivatives of neural ectoderm that form the optic vesicle (i.e., optic stalk, neural retina, and retinal pigment epithelium) and surface ectoderm (i.e., lens and cornea). Eye development in mammals begins with evagination of the optic vesicles toward the lens-competent head surface ectoderm (also called presumptive lens ectoderm). As the optic vesicle contacts surface ectoderm, a series of reciprocal inductive signals elicit formation of the lens placode and subsequent invagination of both lens placode and optic vesicle to form a two-layered optic cup with retinal pigmented epithelium surrounding the retina (reviewed by (Fuhrmann, 2010)). Using conditional gene targeting in mice it was established that *Pax6* is cell-autonomously required for lens placode formation (Ashery-Padan et al., 2000). An evolutionary conserved mechanism was identified by which Pax6 controls the downregulation of multiple genes (such as Sox11) through direct upregulation of miR-204 (Shaham et al., 2013).

In addition to the cell-autonomous role of *Pax6* in the lens compartment *Pax6* appears to be required for lens development also non-autonomously in the optic vesicle (Klimova and Kozmik, 2014). When *Pax6* is eliminated from optic vesicle before its transition to the optic cup then the lens is not formed (Klimova and Kozmik, 2014). Interestingly, Pax6 is required for lens formation only before the transition of optic vesicle into optic cup. Once the lens pit starts to emerge from the lens placode, lens development is no longer dependent on Pax6 being expressed in the neural retina. At the time the lens placode is formed, the dorsal region of the optic vesicle becomes specified to the retina populated with mitotically active retinal progenitor cells (RPCs) (Levine and Green, 2004). Lineage tracing studies have shown that RPCs are multipotent with a single progenitor cell competent to give rise to all retinal neuron and glia cell types (Turner and Cepko, 1987; Holt et al., 1988; Turner et al., 1990). The defining feature of RPCs is co-expression of transcription factors Rx, Pax6, Lhx2, Meis1/Meis2, Six3/Six6, Vsx2, and Hes1, which are expressed prior to the activation of neurogenic program and contribute to the proliferative and retinogenic potential of RPCs (Oliver et al., 1995; Burmeister et al., 1996; Tomita et al., 1996; Mathers et al., 1997; Porter et al., 1997; Marquardt et al., 2001; Liu et al., 2010; Klimova and Kozmik, 2014; Liu and Cvekl, 2017; Diacou et al., 2018; Dupacova et al., 2021). In a defined birth order, RPCs differentiate into seven retinal cell types: retinal ganglion cells, horizontal cells and cone photoreceptors differentiate first, followed by amacrine cells and rod photoreceptors, bipolar cells, and finally Muller glia cells (Young, 1985). As retinogenesis proceeds, RPCs are exposed to the changing environment of extrinsic cues (Cepko, 1999). These, in cooperation with intrinsic factors represented by transcription factors, most prominently of the basic helix-loop-helix (bHLH) and homeodomain class, regulate progenitor proliferation and operate to direct the bias towards particular cell types (reviewed in (Cepko, 1999; Hatakeyama and Kageyama, 2004; Zagozewski et al., 2014)). At the time of neuronal differentiation, the subpopulation of progenitors undergoes transition from the proliferative stage towards the lineage-restricted neurogenic stage, when it withdraws from the cell cycle to take up neuronal or glial fate. The proper balance between the cell cycle exit and re-entry is required to ensure temporal generation of all retinal cell types (reviewed in (Agathocleous and Harris, 2009)).

Teleost fish have become popular to study various aspects of developmental biology and genetics of the eye. Teleost eye shows a high degree of similarity to that of mammals including human (Richardson et al., 2017). As in mammals, the retina of zebrafish and medaka possesses six types of neurons and one type of glia arranged into three nuclear layers. Moreover, in all vertebrates analyzed (mammals and fish included), the generation of neuronal and non-neuronal retinal cell types follows the same stereotyped birth order. Retinal ganglion cells are generated first, whereas bipolar cells and Muller glia are the last cell types to be born (Cepko et al., 1996; Livesey and Cepko, 2001). Although the duration of eye development in human, mouse, chick, zebrafish or medaka is vastly different, the sequence of events, the type of embryonic tissues involved, and spatiotemporal expression of key regulatory genes is remarkably similar. Not only the global eye ‘organ plan’ is largely comparable across vertebrate species, but also the gene regulatory network that is involved in orchestrating eye development is supposed to be mostly conserved (Zhang et al., 2023). Medaka (*Oryzias latipes*) is a small freshwater fish of the family Adrianichthyidae. It is closely related to pufferfish or stickleback and is more distant to the most widely used teleost model organism, zebrafish (*Danio rerio*) (Furutani-Seiki and Wittbrodt, 2004; Signore et al., 2009). Developmental stages of medaka and corresponding morphological characteristics have been described in detail by Iwamatsu (2004). In comparison to zebrafish, medaka embryonic development, including the eye, is slower (Tena et al., 2014). Eye development in medaka is initiated by the specification of the retina anlage in the anterior neural plate at late gastrula stages (stage 15, 16 h post fertilization, hpf). Next the presumptive retina evaginate laterally to form the optic vesicles and contacts the surface ectoderm cells. At the 6-somite stage (stage 21, 28 hpf) the optic cup contains two layers: an inner pseudostratified neuroepithelium from which the neural retina will form, and a thin layer of pigment cells. Retina differentiation is initiated at the 22-somite stage (stage 26, 54 hpf) in the central retina. As in other vertebrates the first neurons to be born are the ganglion cells. The basic helix-loop-helix transcription factor ath5 is first expressed at 54 hpf, marking the onset of ganglion cell differentiation. Morphologically, the three layers of the neural retina appear by 70 hpf (stage 29) and a fully patterned retina is formed by 9 days post fertilization (9 dpf) (Kitambi and Malicki, 2008). Retina differentiation and layer formation in medaka (and zebrafish) progress from the center towards the periphery. The edges of mature fully functional fish retina, the ciliary marginal zone (CMZ), remain undifferentiated and contain retinal progenitor cells (stem cells) that continue to proliferate during the entire adulthood (Perron et al., 1998). Unlike mammals, fish are therefore able to continuously grow their retinas throughout life. In addition to the retinal stem cells present in CMZ there is another population of proliferating cells in postembryonic fish retina. These are Muller glia, which are localized throughout the entire differentiated fish retina. Unlike in mammals, Muller glia in fish is able to produce rod photoreceptors during normal homeostasis and, upon injury, also other neuronal cell types of the retina (Wan and Goldman, 2016; Lust and Wittbrodt, 2018). The gene regulatory networks controlling Müller glia reprogramming upon injury have recently been elucidated (Hoang et al., 2020; Lyu et al., 2023).

Gene and genome duplications are thought to be the driving force of animal evolution. A genome duplication generates paralogous groups of duplicated genes. Being free from selective pressure, paralogous genes undergo neo-functionalization (acquire new function), sub-functionalization (specialize), or one of the paralogs may become extinguished from the genome (Force et al., 1999). After the initial genome duplication, the genomes of different teleost lineages evolved independently. It has become apparent that due to their independent subsequent evolution different fish species show notable differences with respect to the fate of duplicated genes. Medaka and zebrafish are separated from each other by about 110 million years of independent lineage evolution (Wittbrodt et al., 2002; Furutani-Seiki and Wittbrodt, 2004). As a result, medaka possesses a single orthologue of the mammalian *Pax6* gene while zebrafish genome contains two paralogous genes, namely *Pax6.1a* (also referred to as *Pax6a* and *Pax6.1b* (*Pax6b*), respectively (Ravi et al., 2013). Here we investigated the functional role of medaka *Pax6.1* gene in eye development.

## Results

### Expression of Pax6 gene family in medaka embryos and generation of Pax6.1 mutants

**FIGURE 1:**
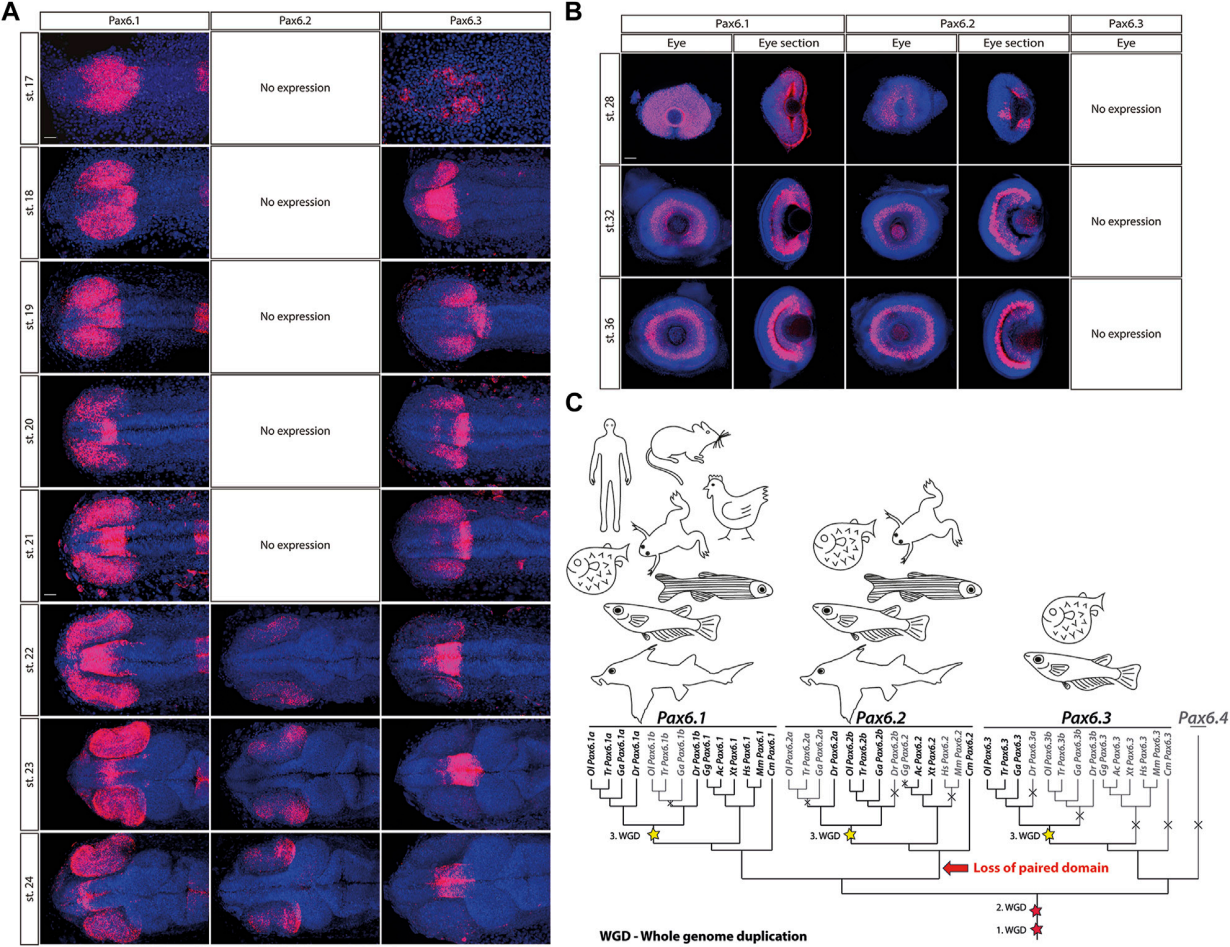
The expression patterns of the *Pax6.1*, *Pax6.2* and *Pax6.3* genes during embryonic development of medaka at stages 17–24 **(A)**, stage 28, 32 and 36 **(B)**. Scale bar: 50 µm. **(C)** The evolutionary trajectories of *Pax6.1*, *Pax6.2*, and *Pax6.3* in vertebrates. Gene losses are depicted by crosses and greyed-out branches/labels. The three WGD events are highlighted with stars. Hs, *Homo sapiens* (human); Mm, *Mus musculus* (mouse); Ga, *Gasterosteus aculeatus* (three-spined stickleback); Gg, *Gallus gallus* (chicken); Ac, *Anolis carolinensis* (lizard); Xt, *Xenopus tropicalis* (frog); Dr, *Danio rerio* (zebrafish); Ol, *Oryzias latipes* (medaka); Tr, *Takifugu rubripes* (fugu); Cm, *Callorhinchus milii* (elephant shark).

The evolutionary history of the *Pax6* gene family in vertebrates has been elucidated by Ravi et al. (2013). As a result of two rounds of vertebrate-specific and one round of teleost-specific whole genome duplication followed by lineage specific gene losses, Acanthopterygians (e.g. medaka, stickleback, pufferfish) retained three genes, namely *Pax6.1*, *Pax6.2*, and *Pax6.3*, respectively. *Pax6.1* and *Pax6.3* genes are structurally similar, and encode both paired domain and homeodomain. In contrast, *Pax6.2* lacks the paired domain, which is a critical DNA-binding domain of Pax family of transcription factors, and thus functionally falls into a large group of paired-type homeodomain proteins. Gene expression analysis in medaka embryos supports previous phylogenetic analysis (Ravi et al., 2013) in assigning *Pax6.1* as a true orthologue of mammalian *Pax6* (Figure 1; (Ravi et al., 2013)). Like its mouse orthologue, *Pax6.1* is strongly expressed throughout the developing optic vesicle from the onset of eye formation. It also appears to be expressed from stage 21 and more conspicuously from stage 22 in the forming lens (Figure 1A). Expression of *Pax6.1* remains high throughout the neural retina and CMZ at later stages when differentiation is initiated (Figure 1B) and coincides with markers of ganglion and amacrine cells in the central retina at stage 32 (Supplementary Figure S1).

In contrast, the eye-specific expression of *Pax6.3* is limited to the posterior part of the optic vesicle at stages 18–22. Next, by stage 23 *Pax6.3* gene becomes sharply downregulated and its expression is completely absent from retina at later developmental stages. The two-color whole mount *in situ* hybridization confirmed that *Pax6.3* expression is clearly distinct from that of *Pax6.1* (Supplementary Figure S2). Finally, paired domain-less *Pax6.2* gene only becomes expressed in developing neural retina from stage 22 onwards (Figures 1A, B).

*Pax6.1, Pax6.2,* and *Pax6.3* genes have distinct evolutionary trajectories (Figure 1C). The subfunctionalization of the two *Pax6.1* paralogs in zebrafish was documented by Kleinjan et al. (2008), where both genes retained seemingly identical and redundant expression in the eye, while the *Pax6.1a* paralog lost its expression in the pancreas. In medaka, the evolutionary trajectory of the *Pax6.1* paralogs following the teleost-specific whole genome duplication led to the loss of one copy.

**FIGURE 2:**
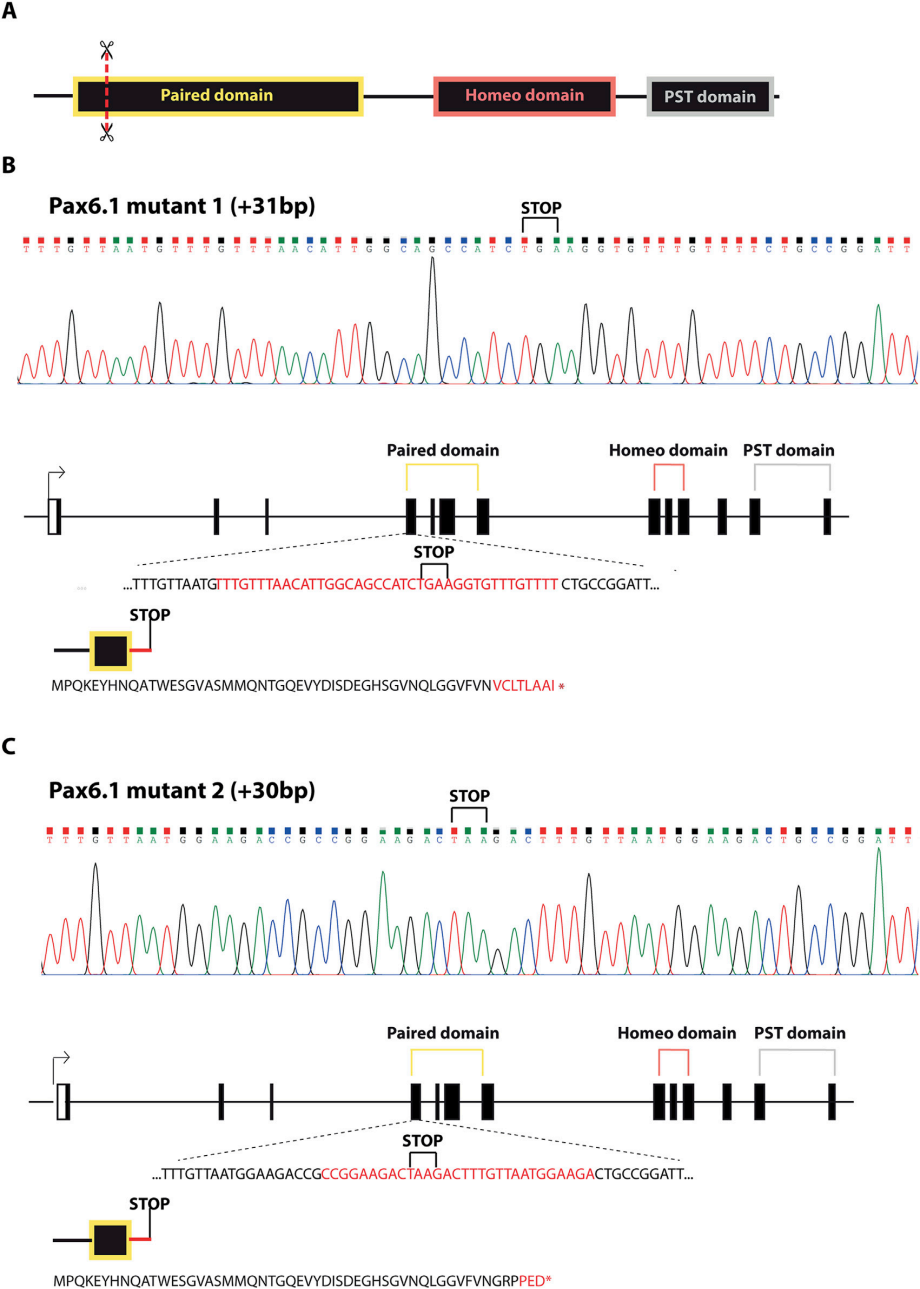
Schematic representation of *Pax6.1* gene editing resulting in the two lines carrying frameshift mutations in the paired domain. **(A)** Schematic diagram of the domain structure of Pax6.1 indicating the position of genome editing tools (scissors). **(B, C)** DNA sequencing and the associated protein sequences for alleles corresponding to Pax6.1 mutant1 and mutant2, respectively.

To determine the functional role of *Pax6.1* gene in eye development we analyzed medaka mutants generated by genome editing. Two independent frameshift alleles of *Pax6.1* were generated by targeting 5′end of the exon encoding the N-terminal half of paired domain thus producing a complete loss-of-function genotypes (designated *Pax6.1* KO mutant 1 and mutant 2; Figure 2). Viable adult homozygotes for *Pax6.1* mutant lines were not recovered (mutants die around hatching) and so the genetic crosses using heterozygotes were established in order to produce embryos for subsequent anatomical and gene expression. To confirm that no functional Pax6.1 paired domain-containing protein product is produced from the genetically manipulated alleles we analyzed RNAs products. It is well established that Pax6 orthologues undergo alternative splicing within paired domain encoding exons leading to either inclusion or exclusion of exon 5a (Kozmik et al., 1997; Fabian et al., 2015). As shown in Supplementary Figure S3, by analyzing RNA products from wild type and mutant alleles we have indeed detected both +5a and −5a variants (+5a variant labeled by asterisk). While the mutant 2 allele produced only the predicted products, the mutant 1 allele generated, in addition to the prediced product, an aberrant variant (designated X in Supplementary Figure S3). DNA sequencing revealed that the mutant1X RNA results from 91bp deletion effectively leading to the frameshift and a truncated Pax6.1 protein.

Combined, phylogenetic and expression data show that medaka *Pax6.1* gene is the true orthologue of *Pax6* in mammals. To study function of medaka *Pax6.1* we produced null alleles suitable for functional analysis.

### *Pax6.1* is required for lens placode induction in medaka

**FIGURE 3:**
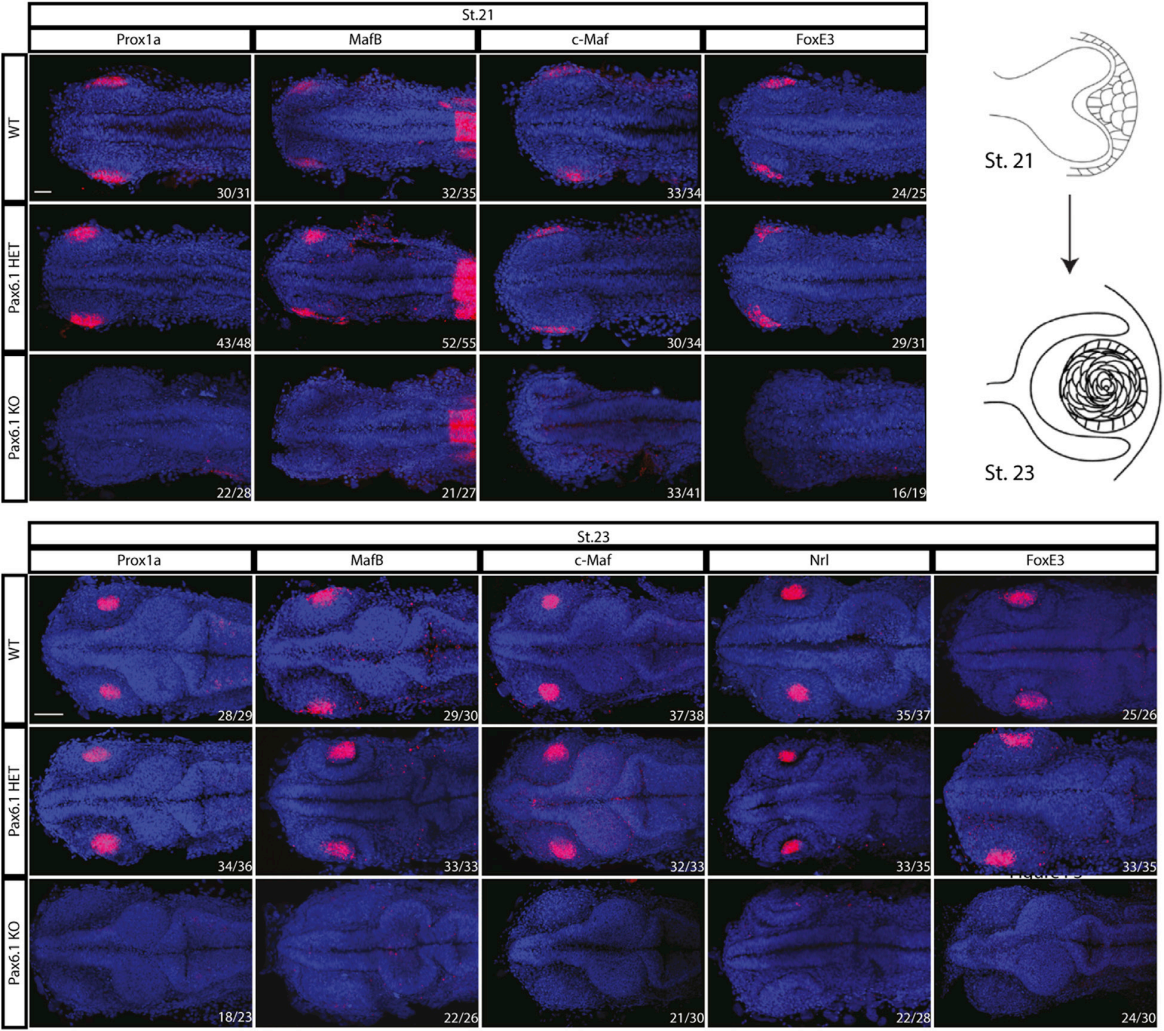
Comparison of the initiation of lens formation by *in situ* hybridization for selected lens markers at stage 21 (*Prox1a*, *MafB*, c-*Maf,* and *FoxE3*) and stage 23 (*Prox1a*, *MafB*, c-*Maf, Nrl,* and *FoxE3*). Signal in the presumptive lens region is present in the wildtype and *Pax6.1* heterozygote but not in the *Pax6.1* homozygote embryos. Scale bar: 50 µm.

We first noted that approximately 25% of stage 28 embryos from *Pax6.1* mutant heterozygote crosses did not contain lenses. Since those embryos were genotyped as *Pax6.1* mutant homozygotes we next aimed to determine if lens induction step was affected in the absence of *Pax6* gene function as is the case in mice (Ashery-Padan et al., 2000). We were unable to detect lens placode marker gene expression prior to stage 21 (Supplementary Figure S4). However, by stage 21 a suite of genes encoding transcription factors implicated in vertebrate lens development (*Prox1a*, *MafB*, *c-Maf*, *FoxE3*) comenced expression in lens placode of wild type and *Pax6.1* mutant heterozygote fish (Pax6.1 HET) but not in *Pax6.1* mutant homozygotes (Pax6.1 KO) (Figure 3). Expression of *Prox1a*, *MafB*, *c-Maf*, *FoxE3*, and *Nrl* remained high in wild type and *Pax6.1* mutant heterozygotes but was not detectable in *Pax6.1* mutant homozygotes at stage 23 (Figure 3) consistent with the appearent absence of lens tissue.

Combined, our data show that *Pax6.1* mutants do not develop ocular lens.

### Ectoderm enhancer (EE) is dispensable for lens development in medaka

**FIGURE 4:**
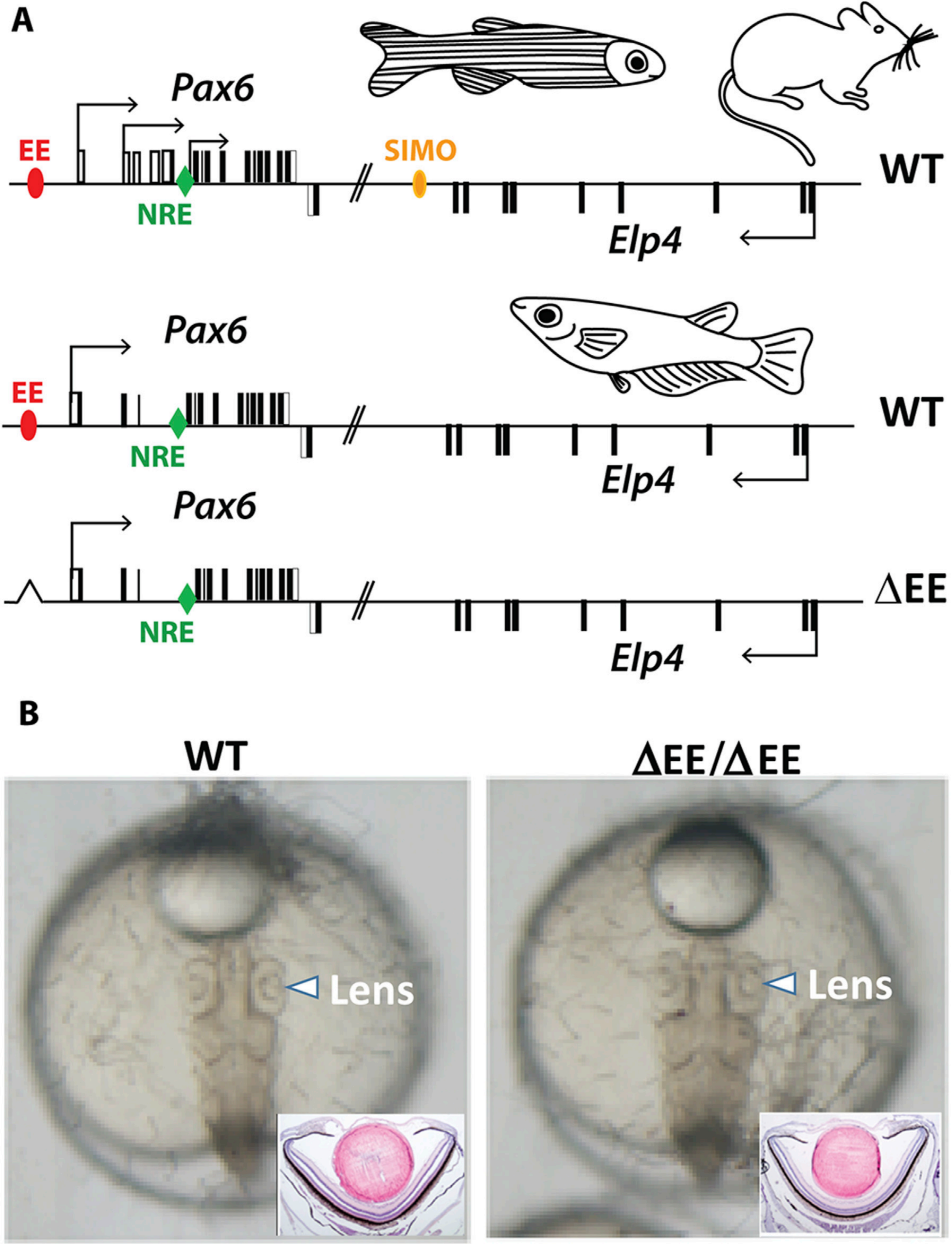
Genome editing of medaka ectoderm enhancer (EE). **(A)** Schematic view of Pax6 locus in mouse, zebrafish, and medaka. The position of the shadow enhancers EE and SIMO is shown by red and yellow ovals, respectively. The position of the evolutionarily conserved retina-specific enhancer NRE (aka α-enhancer) is shown by green rhomb. **(B)** Lens development proceeds normally in the fish containing EE homozygote deletion.

We have previously shown that lens-specific expression in the mouse is achieved by the concerted action of two redundant (shadow) regulatory regions, EE and SIMO enhancers (Antosova et al., 2016). Simulatneous deletion of EE and SIMO phenocopies *Pax6* loss-of-function alleles (Ashery-Padan et al., 2000; Antosova et al., 2016). Although both of those shadow enhancers are evolutionarily conserved in zebrafish (Antosova et al., 2016) we were only able to identify EE but not SIMO in acanthopterygian lineage (medaka, stickleback, pufferfish) indicating either loss of the SIMO enhancer or significant sequence divergence. It is likely that EE plays a more dominant role over SIMO in regulating lens-specific expression of Pax6 in mice as smaller lenses are ocasionally observed in EE but not in SIMO homozygotes (Dimanlig et al., 2001; Antosova et al., 2016). This notion, together with the appearent absence of SIMO prompted us to genetically ablate EE in medaka in order to achieve tissue-specific (lens-restricted) knockout of *Pax6.1*. Unexpectedly, medaka fish carrying a homozygous deletion of EE presented a fully developed lens Figure 4).

Taken together, genetic ablation of EE, the evolutionarily conserved lens-specific enhancer in medaka, does not abrogate lens development.

### *Pax6.1* is required for the differentiation of retinal ganglion cells

**FIGURE 5:**
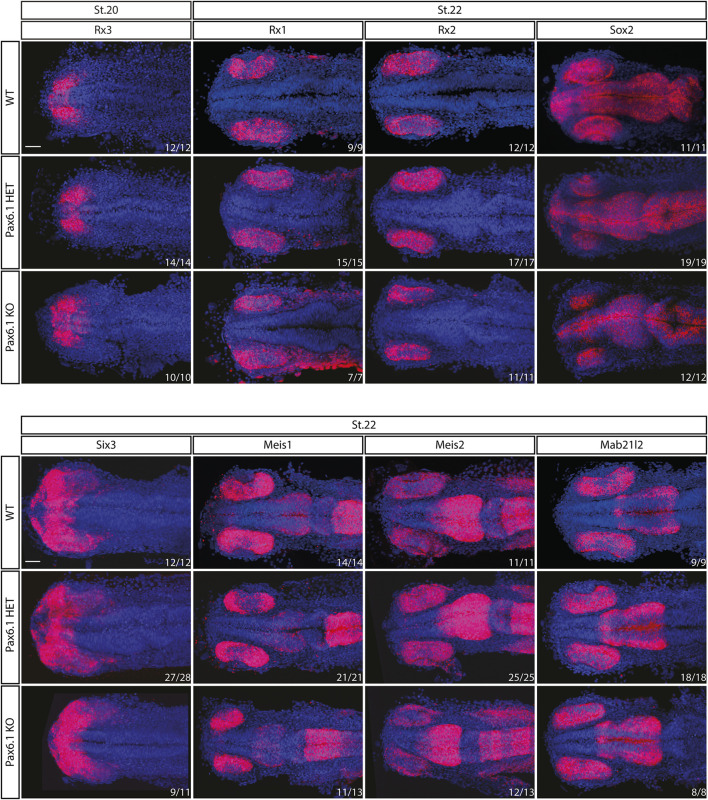
*In situ* hybridization analysis of *Rx3, Rx1, Rx2, Sox2*, *Six3*, *Meis1*, *Meis2,* and *Mab21l2* gene expression during the early retina development (stage 20 and stage 22). The expression pattern of none of the genes is changed in *Pax6.1* homozygote mutant as compared to wildtype or *Pax6.1* heterozygote. Scale bar: 50 µm.

**FIGURE 6:**
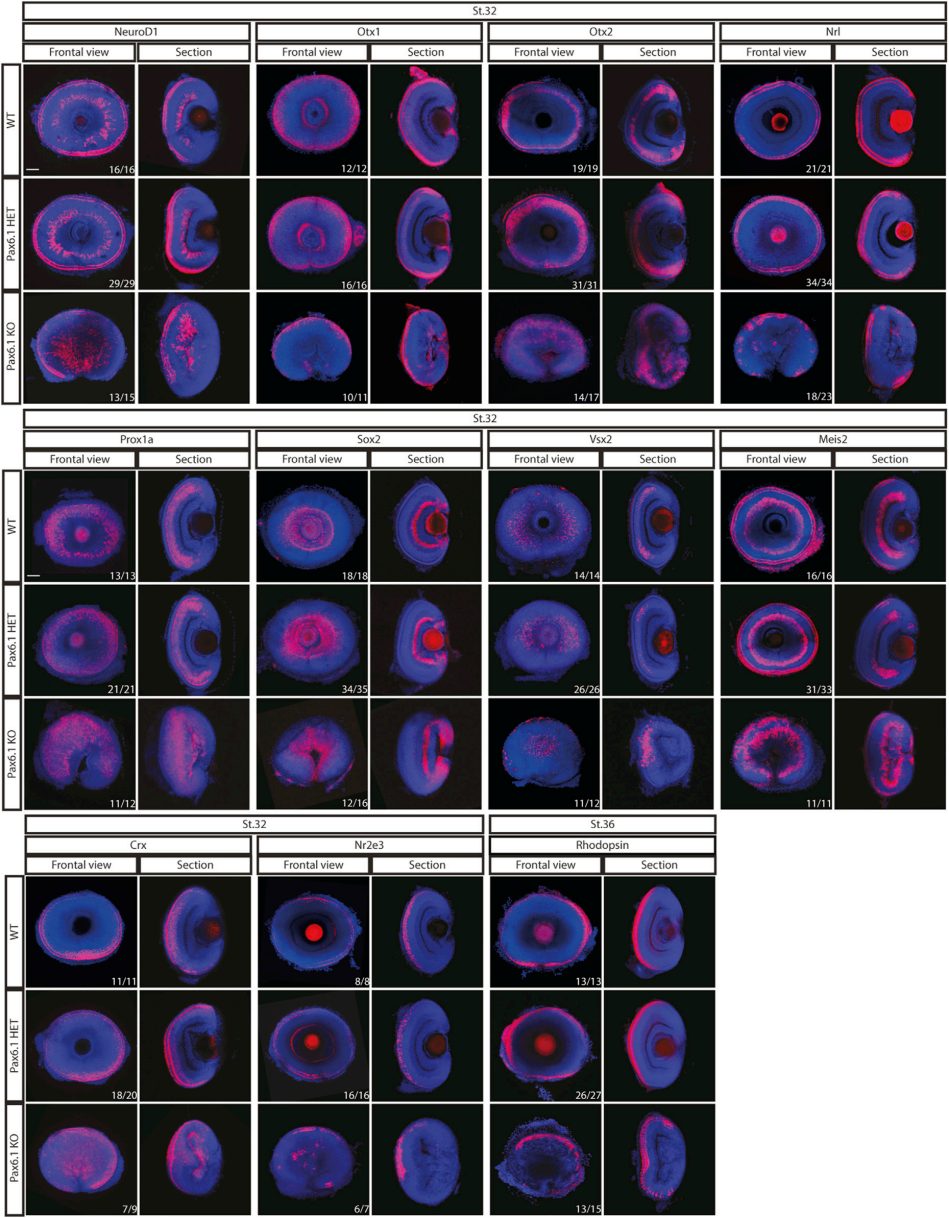
*In situ* hybridization analysis of gene expression in the differentiated retina (stage 32 and stage 36). Comparison of expression patterns of markers for specific retina cell types (*Prox1a*–horizontal cells; *Meis2* – amacrine cells; *Vsx2*-bipolar cells; *Sox2* - Muller glia cells; *Otx1*, *Otx2*, *Nrl*, *Rx2*, *NeuroD1*, *Crx*, *Nr2e3,* and *Rhodopsin -* photoreceptors) in the wild type, *Pax6.1* heterozygotes, and homozygotes, respectively. The expression of markers was not conspicuously altered in the *Pax6.1-*deficient retina as compared to the wild type. Scale bar: 50 µm.

**FIGURE 7:**
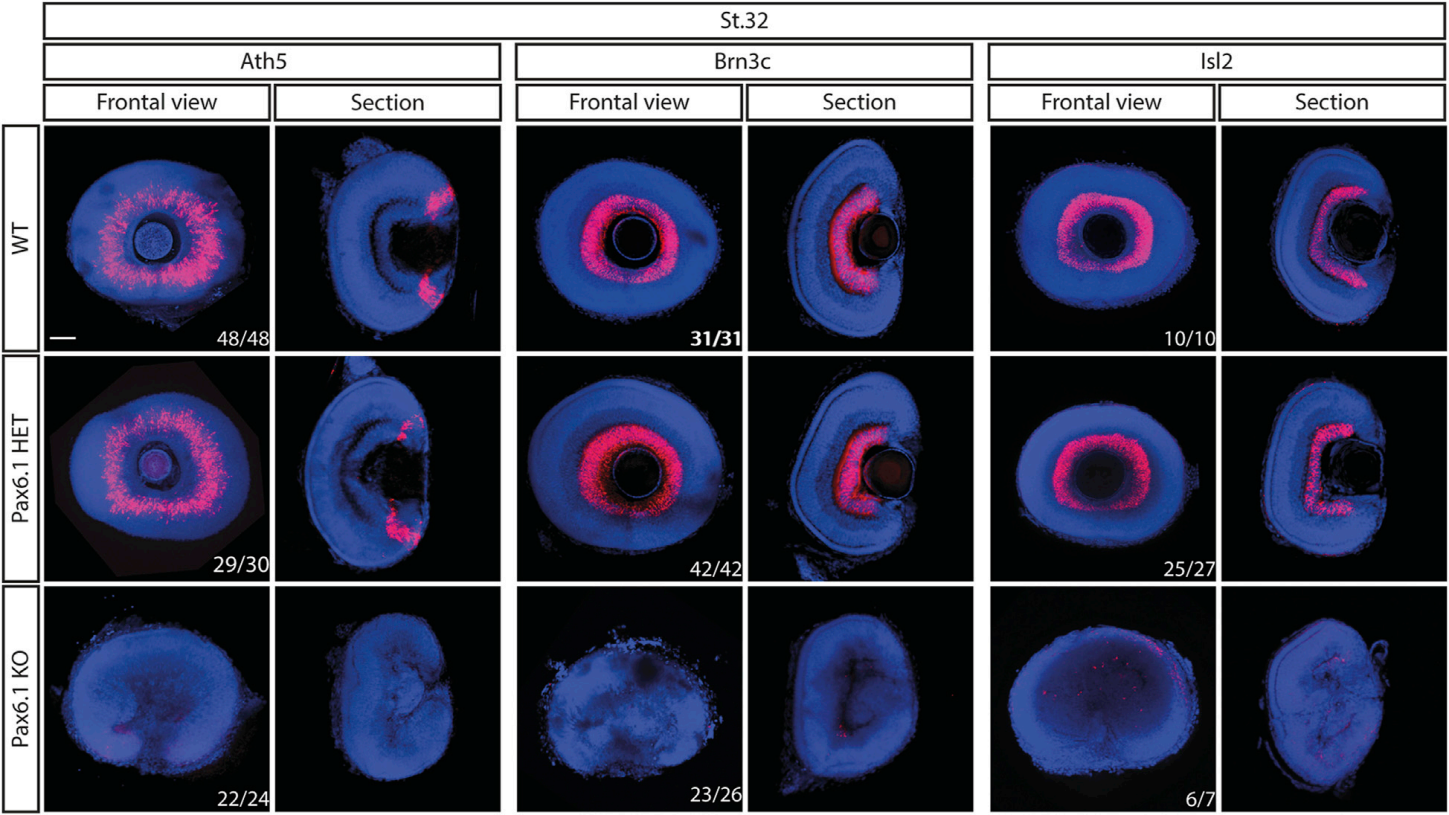
Comparison of expression patterns of retinal ganglion cell markers *Ath5*, *Brn3c*, and *Isl2* in the wild type, *Pax6.1* heterozygotes, and homozygotes at stage 32. None of the retinal ganglion cell markers shown here is expressed in *Pax6.1-*deficient retina Scale bar: 50 µm.

We have previously shown that in mice *Pax6* gene is required cell-autonomously for the expansion of RPCs, and for the differentiation of all retina cell types (Klimova and Kozmik, 2014). Hence we first investigated whether retinal progenitor characteristics were maintained in *Pax6.1* mutants. We assessed the expression of known markers such as *Rx3* at stage 20, as well as *Rx1, Rx2, Sox2, Six3, Meis1, Meis2*, and *Mab21l2* at stage 22. However, the expression of none of these factors was significantly changed (Figure 5) indicating a normal emergence of RPC fate. The proliferative capacity of *Pax6.1*-deficient RPCs as assessed by the phospho-histone H3 marker immunohistochemistry also appeared normal (Supplementary Figure S5). We next tested the differentiation potential of *Pax6.1*-deficient RPCs by the whole-mount *in situ* hybridization at stage 28, stage32, and stage 36 respectively, using a panel of specific markers (Figure 6; Supplementary Figure S6; Supplementary Figure S7). Expression of photoreceptor markers (such as *Otx1*, *Otx2, Crx, Nrl, NeuroD1, Nr2e3*, and *Rhodopsin*) confirmed the presence of this cell type in *Pax6.1* mutant retina (Figure 6). Likewise, the expression of markers typical for horizontal cells (*Prox1a*), amacrine cells (*Meis2*), bipolar cells (*Vsx2*), and Muller glia (*Sox2*) was detected in *Pax6.1* mutant retina at levels comparable to wild type fish (Figure 6). In contrast, retinal ganglion markers *Ath5*, *Brn3c*, and *Isl2* were completely absent from *Pax6.1*-deficient retina at stage 32 (Figure 7). Unlike the situation in mammals, fish retina grows continuously due to retinal stem cells located at CMZ. These cells actively migrate towards the middle part of retina and differentiate into any retinal cell type. To exclude the formal possibility that the lack of a more profound phenotype in Pax6.1-deficient retina is due to CMZ-derived differentiation program we analyzed specific markers in stage 28, i.e. before the cells from CMZ start to migrate. However, marker gene expression at stage 28 corroborated our conclusion that retinal ganglion cell marker is the only affected one when wild type and Pax6.1-deficient retinae are compared (Supplementary Figure S7).

Taken together, these results indicate that the overall differentiation potential of Pax6.1-deficient RPCs is not severely compromised, and the only cell type affected by the absence of Pax6.1 transcription factor are retinal ganglion cells.

### Mild phenotype in Pax6.1-deficient retina is not due to the compensatory effects caused by *Pax6.3* paralogue

**FIGURE 8:**
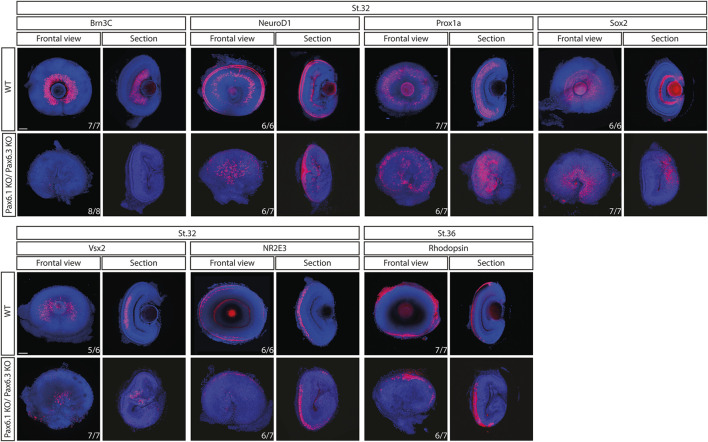
Comparison of expression patterns of *Brn3c*, *NeuroD1*, *Prox1a*, *Sox2*, *Vsx2*, *Nr2e3,* and *Rhodopsin* marker genes in the wild type and *Pax6.1*/*Pax6.3* double homozygote mutants. The retinal ganglion cell-specific expression of *Brn3c* is lost while the expression of the remaining markers is preserved in *Pax6.1*/*Pax6.3*-deficient retina. Scale bar: 50 µm.

*Pax6.1* and *Pax6.3* paralogues are structurally similar and encode transcription factors with similar properties when tested using Pax-responsive luciferase reporter gene *in vitro* (Supplementary Figure S8). To determine, if the deletion of *Pax6.1* gene caused compensatory upregulation of *Pax6.3* expression we analyzed its expression from stage 18 through stage 24. As shown in Supplementary Figure S9 the expression of *Pax6.3* was not enhanced but rather reduced in the *Pax6.1*-deficient retina as compared to the wild type. To examine a possible genetic redundancy of *Pax6.1* and *Pax6.3* we used genome editing to mutagenize *Pax6.3* gene (Supplementary Figure S10). We next analyzed a general morphology (Supplementary Figure S11) and marker gene expression in the developing retina of single *Pax6.3* mutant (Supplementary Figure S12) and of *Pax6.1/Pax6.3* double mutant (Figure 8). We have observed that the lens was present in the *Pax6.3* single mutants and that the overal retina size has not changed in *Pax6.1/Pax6.3* double mutants as compared to the *Pax6.1* single mutants (Supplementary Figure S11). All markers interrogated in the *Pax6.3* mutant retina were expressed including retinal ganglion cell-specific *Brn3c* (Supplementary Figure S12). Finally, retinal ganglion cells were the only cell type conspicuously absent in the *Pax6.1/Pax6.3* double mutant retina (Figure 8).

Combined, our data strongly suggest that the relatively mild retina phenotype observed in Pax6.1 medaka mutant is not due to (i) compensatory mechanisms occuring at the transcriptional level or (ii) the genetic redundancy.

## Discussion

**FIGURE 9:**
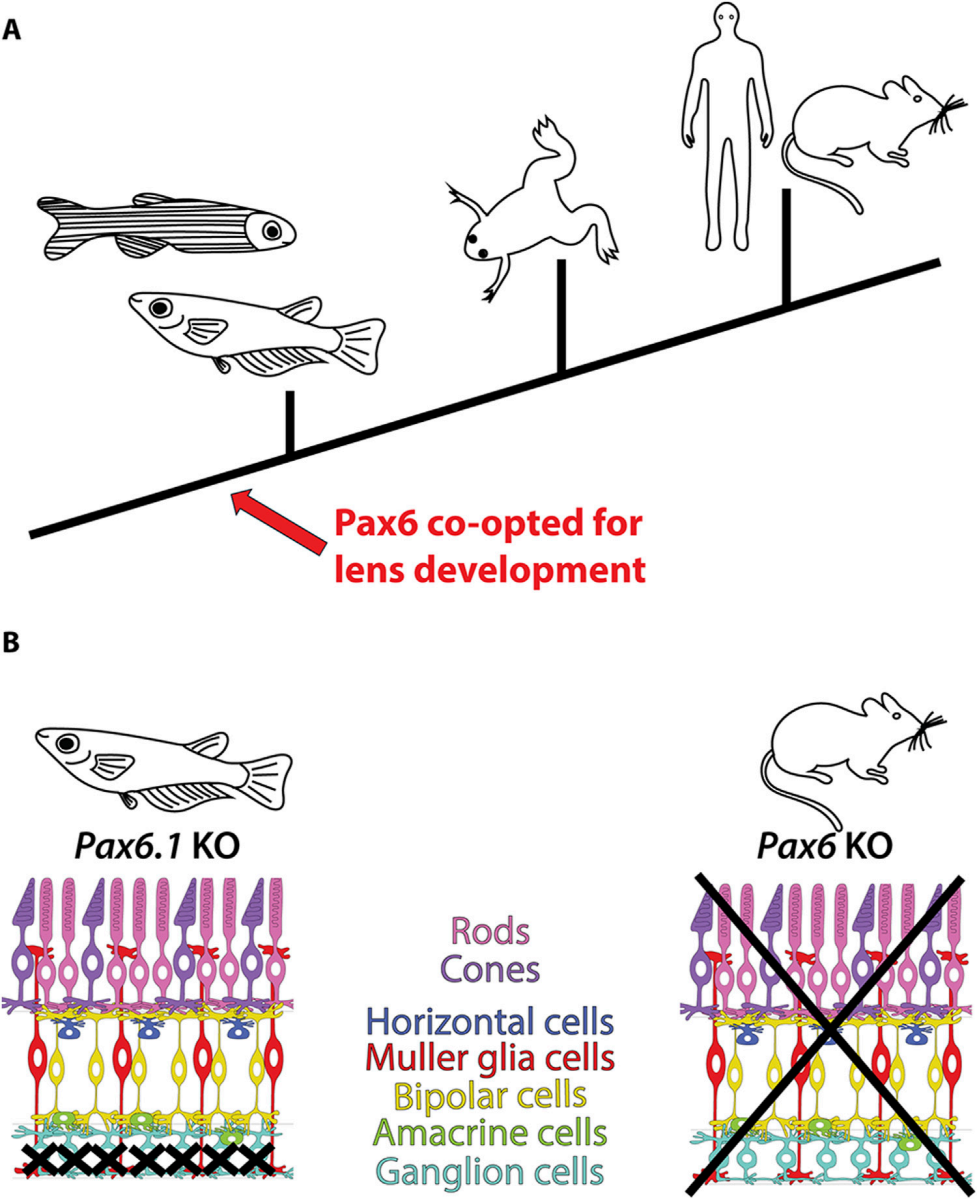
Conserved and divergent roles of Pax6 in vertebrate eye development. **(A)** The lens-specific function of Pax6 seems to be evolutionarily conserved among jawed vertebrates, as evidenced by data from fish (this study and (Kleinjan et al., 2008; Takamiya et al., 2020)), frog (Nakayama et al., 2015), mouse (Hill et al., 1991; Ashery-Padan et al., 2000; Antosova et al., 2016), and human (Glaser et al., 1992). **(B)** Divergent roles of *Pax6* in retinal development in mice and medaka fish. In mice, Pax6 is essential for the formation of all retinal cell types (Klimova and Kozmik, 2014), whereas in medaka fish, only retinal ganglion cells are critically dependent on Pax6 gene function (this study).

Our genetic study, capitalizing on the reduced *Pax6* gene complement in medaka fish, illuminates conserved and divergent roles of *Pax6* orthologues in vertebrate eye development. Somewhat counterintuitively we found that lens-specific role of Pax6 is more evolutionarily conserved among vertebrates than the role of *Pax6* in retina development (Figure 9A). Ocular lens is an upgrade of the animal visual system that occured multiple times during animal evolution (Jonasova and Kozmik, 2008). The vertebrate camera eyes have been acquired independently to other phyla with image-forming vision. The lens acquisition seems to have occurred in the earliest period of the vertebrate lineage, because the fossil stem vertebrates appear to have possessed eyes with lenses (Shu et al., 1999; Shu et al., 2003; Morris and Caron, 2014). Extant representatives of basal vertebrate lineages (cyclostomes) either do not have lenses due to the presumed degeneration as in hagfish, or in the case of lampreys possess flattened immature lenses at the larval stages that develop into fully functional lens only after metamorphosis (Suzuki and Grillner, 2018).

The nature of the possible conserved role of *Pax6* in vertebrate lens formation is currently enigmatic. For example, there is a clear distinction in lens morphogenesis among present-day vertebrates - lens development procceeds via delamination in fish but through invagination in mammals or birds. *Pax6* appears to be a critical gene for shroom-mediated lens invagination of mammalian lens (Plageman et al., 2010), a role clearly not needed in fish lens development. Furthermore, the well-established role of *Pax6* in lens crystallin gene regulation (Cvekl and Piatigorsky, 1996; Cvekl et al., 2015; Cvekl et al., 2017) represents the case of convergent evolution since the crystallin genes are often taxon specific, and Pax6 transcription factor has been therefore independently co-opted in different lineages.

Medaka and zebrafish lineages have undergone an estimated 110 million years of independent evolution (Wittbrodt et al., 2002; Furutani-Seiki and Wittbrodt, 2004) which in medaka apparently lead to the loss of one of the *Pax6.1* copies produced by teleost-specific whole genome duplication. The presence of two *Pax6.1* paralogous in zebrafish genome, *Pax6.1a* and *Pax6.1b* hampers genetic analysis. Furthermore, the duplicated zebrafish *Pax6.1* genes have subfunctionalized by cis-regulatory divergence (Kleinjan et al., 2008) which further complicates interpretation of *Pax6* gene function in the main fish model organism. Missense mutation (L244P) in the homeodomain of zebrafish *Pax6.1b* gene in *sunrise* mutant causes anterior chamber defects (Kleinjan et al., 2008; Takamiya et al., 2015). However, the true genetic loss-of function mutants of both *Pax6a* and *Pax6b* in zebrafish have not been described. However, Takamiya et al. (2020) used *Pax6.1a*/*Pax6.1b* ^*sunrise*^ double mutants to demonstrate the role of *Pax6.1* gene in the control of neural crest cells during development of the anterior segment. The severe anterior segment dysgenesis phenotype in *Pax6.1a*/*Pax6.1b* ^*sunrise*^ homozygote mutants was characterized by the absence of the lens, corneal endothelium, and vasculature. The iridocorneal angle were misssing in the compound *Pax6.1a*/*Pax6.1b* ^*sunrise*^ mutants and the eye structure was filled with abnormal ocular mesenchymal cells. The results indicated that *Pax6.1* paralogs facilitate the expression of guidance molecules in the optic cup and its surrounding mesenchymal cells. Some defects in the anterior segment of *Pax6.1a*/*Pax6.1b* ^*sunrise*^ homozygote mutants were at least partially due to the absence of the lens, which serves as a crucial source of further signaling molecules, such as TGFβ. Severe anterior segment dysgenesis in *Pax6.1a*/*Pax6.1b* ^*sunrise*^ homozygous mutants could in fact be alleviated by transplantation of a wild type lens. We have observed that the lack of lens in Pax6.1 medaka mutants causes a structural collapse of the anterior chamber (Supplementary Figure S13). It is of note that even though a full-length Pax6.1b protein is made from *sunrise* allele, the compound *Pax6.1a*/*Pax6.1b* ^*sunrise*^ homozygotes did not appear to possess a conspicuous ganglion cell layer indicating an extreme sensitivity of this cell type to *Pax6* gene function.

Previous study describing the loss of lens structure in medaka *Pax6.1* mutant lacked molecular characterization of the observed phenotype such as marker gene expression or description of the onset of the defect (Pan et al., 2023). Likewise, no characterization of the retina tissue in *Pax6.1* mutant medaka was performed (Pan et al., 2023). We found that lens development in *Pax6.1* mutant medaka is arrested at the onset of lens specification since even the earliest lens markers that we used were not expressed in the homozygote mutant. It is well established that *Pax6* is required cell autonomously for lens development in mice (Ashery-Padan et al., 2000; Antosova et al., 2016). In addition, it was shown using tissue-specific ablation in mice that expression of *Pax6* in the retina compartment at the optic vesicle stage is required for lens placode induction and subsequent lens development (Klimova and Kozmik, 2014).

Conditional ablation of genes using Cre/loxP methodology used routinely in the mouse model is not available in medaka. Hence, realizing that SIMO shadow enhancer is not present in medaka (and in other Acanthopterygii fish), and EE appears to be the sole lens placode enhancer of *Pax6.1* gene, we attempted to generate lens-specific knockout of *Pax6.1* by deleting EE enhancer region. This experiment would allow to bypass lethality of *Pax6.1* whole-body knockout, and would help to define if *Pax6.1* is required for lens induction in fish in a cell-autonomous manner. The lack of lens phenotype in EE mutant medaka has two possible explanations that are not necesarilly mutually exclusive. First of all, it is possible that retina-derived *Pax6.1* is required for lens induction in fish as is the case in the mouse (Klimova and Kozmik, 2014). Another possibility is that another shadow enhancer evolved within Acanthopterygii fish to replace SIMO which has deteriorated.

The basic cellular composition of retina of vertebrates is highly conserved (Lamb, 2013). The adult lamprey, a representative of Agnathans, already contains all types of retinal cells found in jawed vertebrates, distributed into three main nuclear layers and two plexiform layers (Lamb, 2013; Suzuki and Grillner, 2018). Moreover, in all vertebrates analyzed so far the generation of retinal cell types follows the same stereotyped birth order. Since the spatiotemporal expression of the key regulatory genes is remarkably similar among model vertebrates, it is generally assumed that the gene regulatory networks involved in the orchestrating eye development may be largely conserved (Zhang et al., 2023). Given the prominent role of *Pax6* in mouse retina development (Marquardt et al., 2001; Klimova and Kozmik, 2014) the relatively mild phenotype observed in medaka *Pax6.1* mutant is intriguing. We have previously found that the first manifestation of abnormal retina development in *Pax6* mutant mice is hypocelularity caused by the loss of RPC proliferation and extended cell cycle length leading to the complete absence of retina tissue by birth (Klimova and Kozmik, 2014). This is clearly distinct from the situation in the medaka fish (Figure 9B). Based on phospho-histone H3 immunostaining *Pax6.1*-deficient RPCs appear to proliferate normally, and as a result the relatively normal size retina is present in the *Pax6.1* mutant larvae. The most striking result of our study was the strict dependance of the Ath5/Brn3c retinal ganglion cell lineage on *Pax6.1* function combined with the fact that differentiation into all other retinal cell types was able to procceed in *Pax6.1* mutants. It is worth noting that transcriptional regulation of *Ath5* in retinal ganglion cells is so far the best example of the evolutionarily conserved mechanism by which *Pax6* operates in the vertebrate retina (Riesenberg et al., 2009; Willardsen et al., 2009). We consider it unlikely that the absence of an effect on non-RGC cell differentiation in *Pax6.1* mutant is due to redundancy with *Pax6.2* gene, given (i) its limited expression pattern and (ii) the absence of the paired domain, which is crucial for retinal differentiation in mice. Nonetheless, the potential redundant roles of *Pax6.2* and *Pax6.1* in medaka retina development merit further investigation. It remains to be determined what adaptive changes were aquired in the teleost and mammalian lineages that are responsible for the distinct requirements for *Pax6* function in RPC expansion and execution of the complete retinal differentiation program. In *Xenopus*, mutations producing truncated Pax6 proteins disrupt forebrain regionalization but do not entirely eliminate eyes. Instead, they result in the development of eye-like structures lacking lenses (Nakayama et al., 2015). It was hypothesized by Nakayama et al. that an additional *Pax6* gene (*Pax6.2*) plays a role in mitigating the degree of the retinal phenotype. Given the results of our study, it is however plausible that *Pax6* gene in *Xenopus* is to some extent not needed for normal retina development indicating that the strict requirement for *Pax6* in retinogenesis arose only after the split of the amphibians from amniots.

## Materials and methods

### Animal husbandry

*Oryzias latipes* (medaka) embryos of the Cab inbred strain (Loosli et al., 2000) were used for all experiments. Embryos were collected daily immediately after spawning. Embryonic stages were determined according to Iwamatsu (2004). Housing of animals and *in vivo* experiments were performed after approval by the Animal Care Committee of the Institute of Molecular Genetics (study ID#84/2014 and ID#14/2017) and in compliance with the European Communities Council Directive of 24 November 1986 (86/609/EEC).

### Genome editing

TALEN- and CRISPR-based tools were designed and prepared as described previously (Antosova et al., 2016). Polyadenylated TALEN mRNA was prepared using mMESSAGE mMACHINE T7 ULTRA Kit (Ambion) and was injected into one-cell stage medaka. Oligonucleotides used to make sgRNA constructs were cloned into pT7-gRNA (pT7-gRNA was a gift from Wenbiao Chen, Addgene plasmid # 46759). Cas9 mRNA was prepared using mMESSAGE mMACHINE T7 ULTRA Kit (Ambion) using plasmid pCS2-nCas9n (pCS2-nCas9n was a gift from Wenbiao Chen, Addgene plasmid # 47929). The sgRNAs were transcribed using MEGAshortscript kit (Ambion). A mixture of Cas9 mRNA (100 ng/μL) and specific sgRNAs (25 ng/μL each) was injected into one-cell stage medaka. Pax6.1 and Pax6.3 TALENs targeted the sequence TTG​GTG​GCG​TGT​TTG​TTA​Atg​gaa​gac​cgc​tgc​cGG​ATT​CCA​CCA​GGC​AGA​AAA and TGG​GAG​ACC​TCT​GCC​CGA​CTc​cac​cag​gca​gaa​gaT​CGT​GGA​GCT​GGC​CCA​CA, respectively. Pax6.1 sgRNA used to generate line 2 targeted the sequence TGT​TAA​TGG​AAG​ACC​GCT​GCCGG (PAM sequence underlined). Medaka ectoderm enhancer (EE) was deleted using sgRNAs targeting the sequences CGA​ACT​GCA​TCT​GAA​AGT​GCAGG and TAA​TGT​CTC​GAT​CCA​GGG​CCAGG (PAM sequence underlined). The injecting setup was as follows: pressure injector Femtojet (Eppendorf), micromanipulator TransferMan NK (Eppendorf), borosilicate glass capillaries (GC100F10, Harward Apparatus), stereomicroscopes (Olympus SZX7, SZX9). The mature F0 fish were crossed with wild-type fish, and their F1 progeny was assayed for mutations by DNA sequencing. Stable lines of mutagenized fish were established and the subsequent generations were genotyped by PCR. To identify transcripts generated from mutated Pax6.1 alleles the total RNA was isolated using TRIZOL reagent from eye-containing head region of medaka larvae. The single stranded cDNA produced by SuperScript VILO cDNA synthesis kit was subjected to PCR amplification using forward primer ACC​ACA​GGC​GAA​AGC​CTA​CAT located in the 5′UTR and reverse primers Rev1 ATC​TTG​CTC​ACG​CAG​CCG​TT or Rev2 CTG​TCC​TGG​CAC​TGA​TGT​T. The products were cloned into TOPO vector and sequenced.

### Whole-mount RNA *in situ* hybridization

Embryos were fixed overnight at 21°C with fixative solution (4% formaldehyde/PBS + 0.1% Tween), dechorionated and stored in methanol at −20°C. During the experiment, samples were rehydrated and treated with Proteinase K to increase penetration (timing was adjusted according to embryonic stage). After refixation with fixative solution, embryos were further processed for overnight hybridization with digoxigenin (DIG) and/or fluorescein (FITC) labelled antisense riboprobes on 65°C. Next day, samples were incubated with anti-DIG or FITC-Fab fragments (Roche) conjugated with alkaline phosphatase or peroxidase, respectively. The coloring reaction was carried out by either VectorBlue (VECTOR Laboratories) or TSA™ Plus Fluorescein System (PerkinElmer). All samples were stained with DAPI and afterward mounted in 86% glycerol/1.5% low gelling point agarose (Serva) for imaging or 4% low gelling point agarose (Serva) for vibratome sectioning.

### Immunohistochemistry

Embryos were fixed overnight at 21°C with fixative solution (4% formaldehyde/PBS + 0.1% Tween) and afterwards dechorionated. Dechorionation was followed by immediate whole-mount immunohistochemistry procedure: Embryos were treated with ice-cold acetone for 7 min, afterwards washed with PBT (PBS + 0.1% Tween) and blocked overnight at 4°C with the Phosphorylated histone H3 (PH3) antibody (Merck) diluted in 10% BSA/PBS + 0.1% Tween (1:500). Next morning, samples were washed several times with PBT and incubated with the Alexa647 antibody (ThermoFisher) for 1.5 h. Subsequently, samples were stained with DAPI and sectioned on a vibratome machine (Leica). Sectioned samples were assessed by light microscopy (high-speed confocal dragonfly spinning disc microscope (Andor)). Total number of cells (stained by DAPI) as well as the number of proliferating cells (stained by PH3) in the retina of 8 wild type and 8 Pax6.1 knock out embryos was calculated manually using ImageJ software (Schindelin et al., 2012). A percentage score was obtained by dividing the number of proliferating cells by the total number of cells for each sample.

### Cell nucleus staining

Embryos at selected stages were fixed overnight at 21°C with fixative solution (4% formaldehyde/PBS + 0.1% Tween) and afterwards dechorionated. Cell nucleus staining was achieved by overnight DAPI (Roche, 1:1,000) staining. Stained embryos were stored in 86% glycerol or embedded in 4% low gelling point agarose (Serva).

### Vibratome sectioning

Embryos after the *in situ* hybridization, immunohistochemistry staining or cell nucleus (DAPI) staining were sectioned using a vibratome machine (Leica). 50 µm thick sections were obtained and mounted in 86% glycerol on slides.

### Imaging

All samples were photographed on a high-speed confocal dragonfly spinning disc microscope (Andor). Pictures were processed by ImageJ software (Schindelin et al., 2012).

### Reporter gene assays

The cell culture and transient cell transfection was performed as previously described (Klimova et al., 2015). CMV-based expression vectors encoding either Pax6.1, Pax6.2, or Pax6.3 were co-transfected with Pax6-resposive reporter gene (−350GluLuc (Schwaninger et al., 1993)) and the β-galactosidase expression plasmid serving to normalize the transfection efficiency. Graph and statistical analysis of triplicate biological assays were generated in GraphPad Prism software.

## Data Availability

The raw data supporting the conclusions of this article will be made available by the authors, without undue reservation.
