# Supplementary material for: Genetic analysis of medaka fish illuminates conserved and divergent roles of Pax6 in vertebrate eye development
Source: Front Cell Dev Biol. 2024 Oct 24;12:1448773. doi: 10.3389/fcell.2024.1448773 (PMC11541176; doi:10.3389/fcell.2024.1448773)
Supplement: Supplementary file 1 [file DataSheet1.PDF]

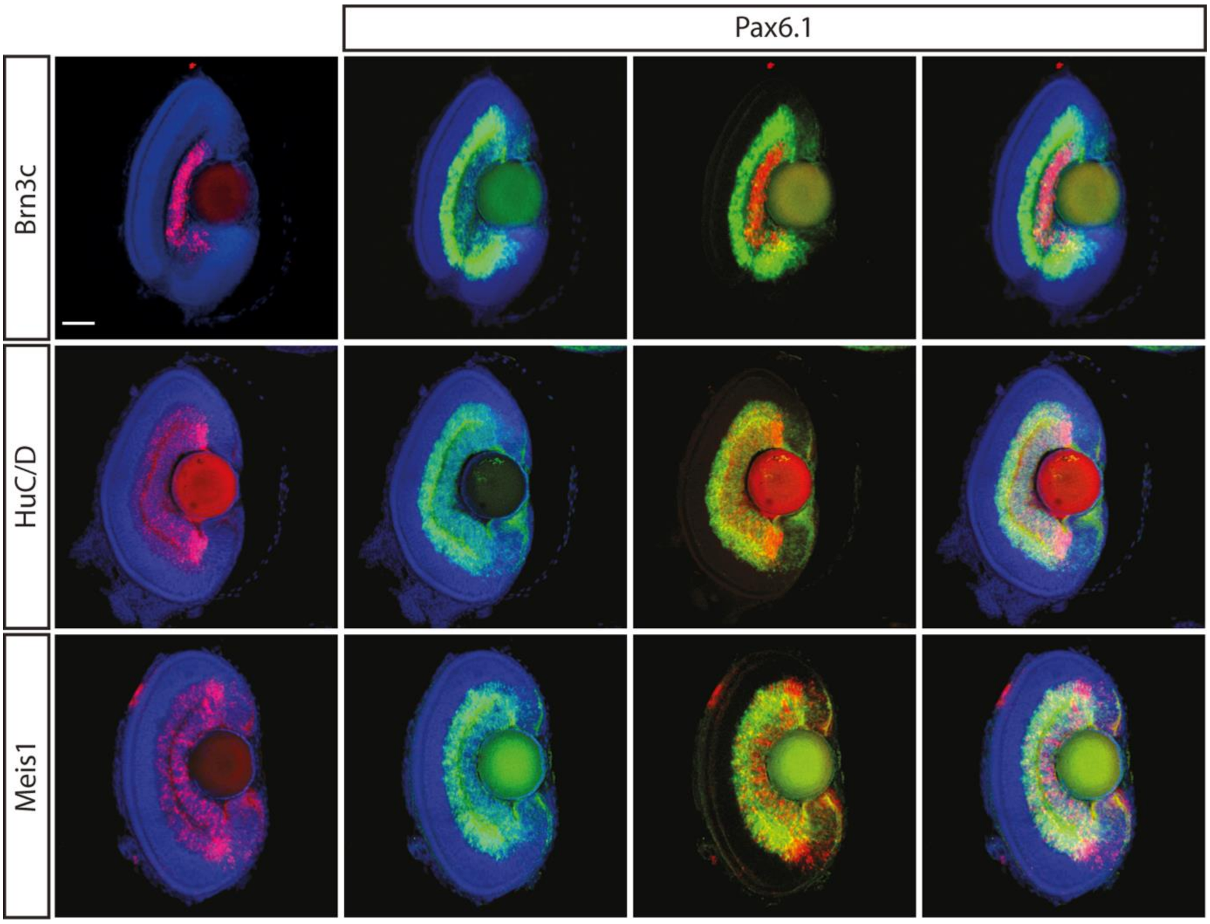

Supplementary Figure 1. Double in situ hybridization at stage 32 for *Pax6.1* and *Brn3c*, *HuC/D*, *Meis1*, respectively. Staining shows the co-expression of *Pax6.1* with selected genes in ganglion and amacrine cell. Scale bar: 50µm

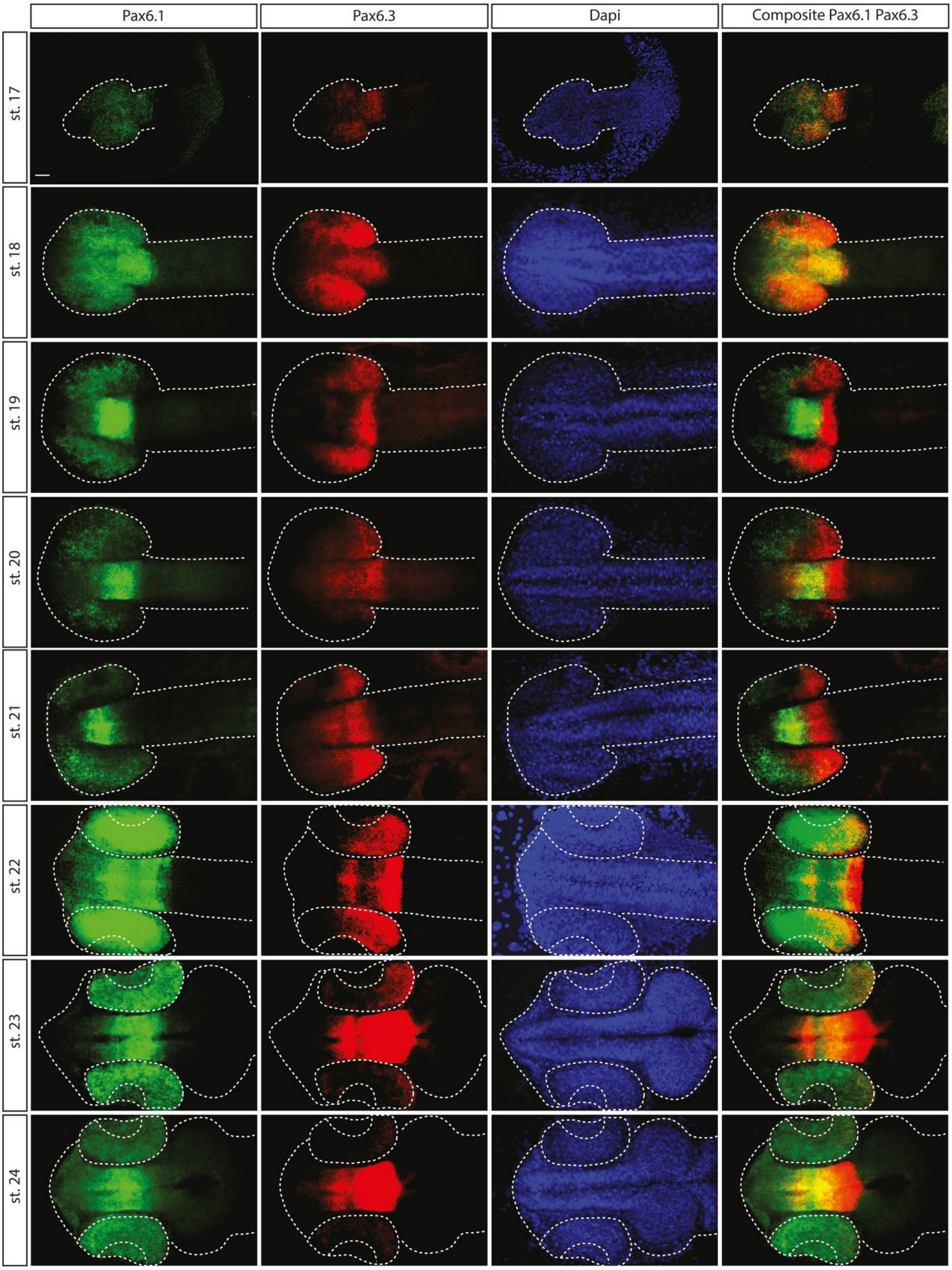

Supplementary Figure 2. Double in situ hybridization for *Pax6.1* and *Pax6.3* genes during the early stages of medaka eye development. *Pax6.1* and *Pax6.3* are co-expressed in the brain and the developing eye area. Scale bar: 50µm

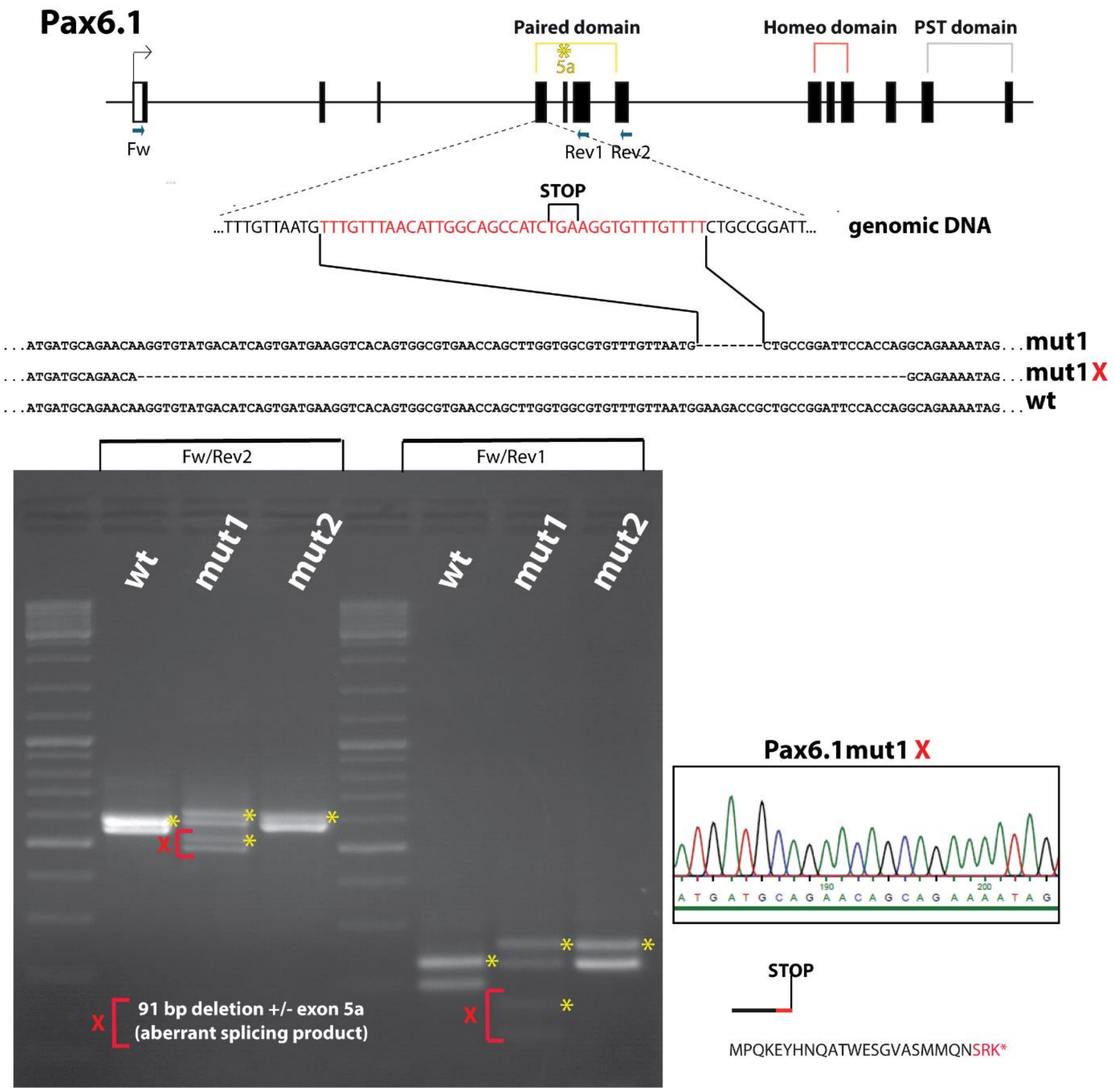

Supplementary Figure 3. Schematic representation of *Pax6.1* gene editing producing mutant line 1. An insertion of a 39bp DNA fragment was accompanied by the deletion of an 8bp sequence from the *Pax6.1* locus thus resulting in the net insertion of +31bp in the genomic DNA. The yellow asterisks indicate variants containing the alternatively spliced exon 5a. In addition to the predicted spliced products, an aberrant variant was produced in mutant line 1 leading to a frameshift and a truncated Pax6.1 protein. Only the predicted variants were produced in mutant line 2.

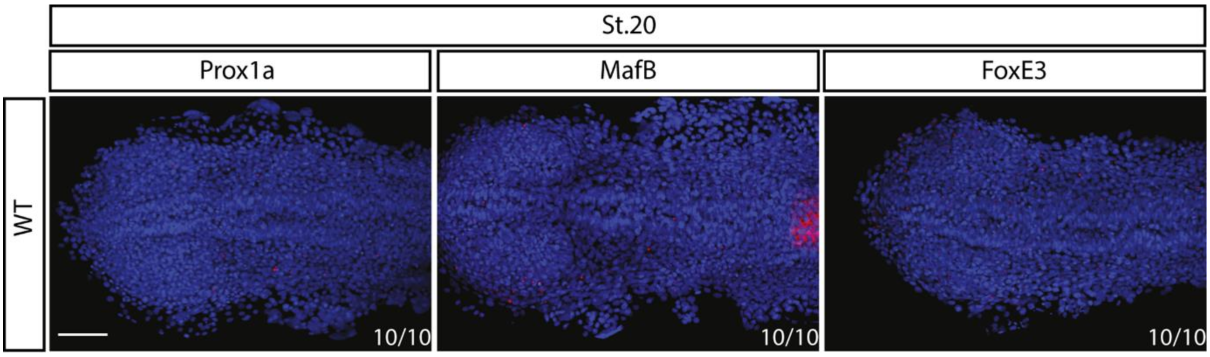

Supplementary Figure 4. No expression of lens markers (*Prox1a*, *MafB*, and *FoxE3*) is detected at the embryonic stage 20**.** Scale bar: 50µm

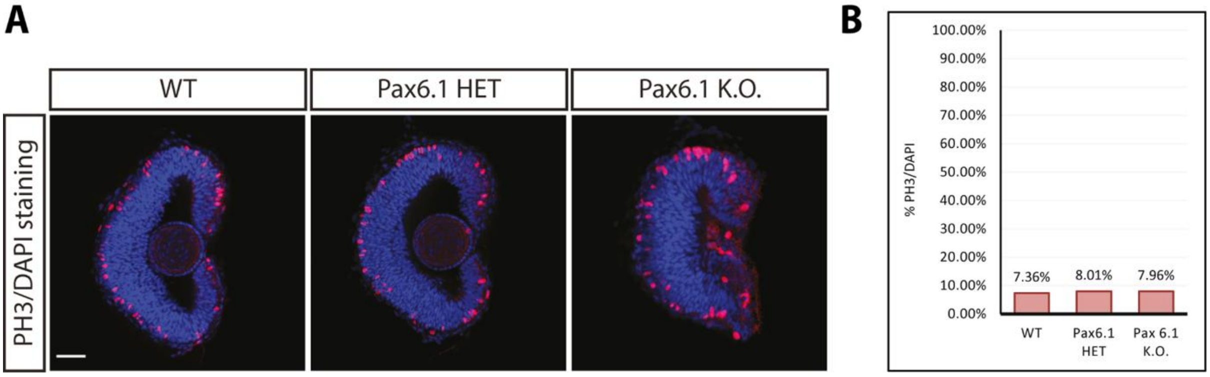

Supplementary Figure 5. Immunohistochemistry staining for phosphorylated histone H3 in wildtype and *Pax6.1*-deficient retina **(A)**. Quantitative analysis of the percentage of PH3 positive cells in the WT, heterozygote and homozygote retina showing no significant difference in the number of proliferating cells **(B)**. Scale bar: 50μm

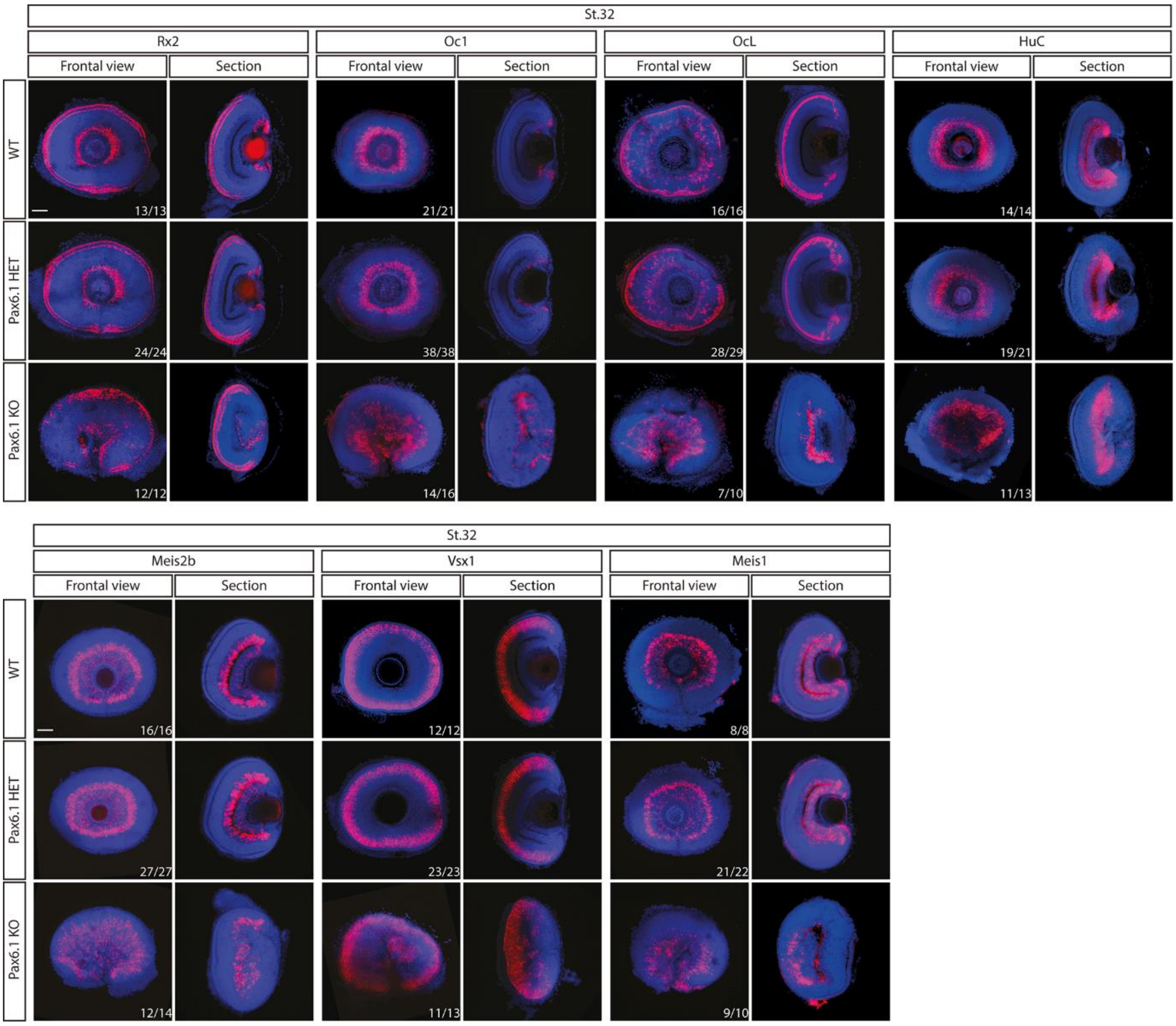

Supplementary Figure 6. Comparison of the expression patterns of selected genes (*Rx2, Oc1, OcL, HuC/D, Meis1, Meis2b, Vsx1*) in the retina between WT, *Pax6.1* heterozygote and homozygote at the stage 32. No changes in expression patterns were observed. Scale bar: 50µm

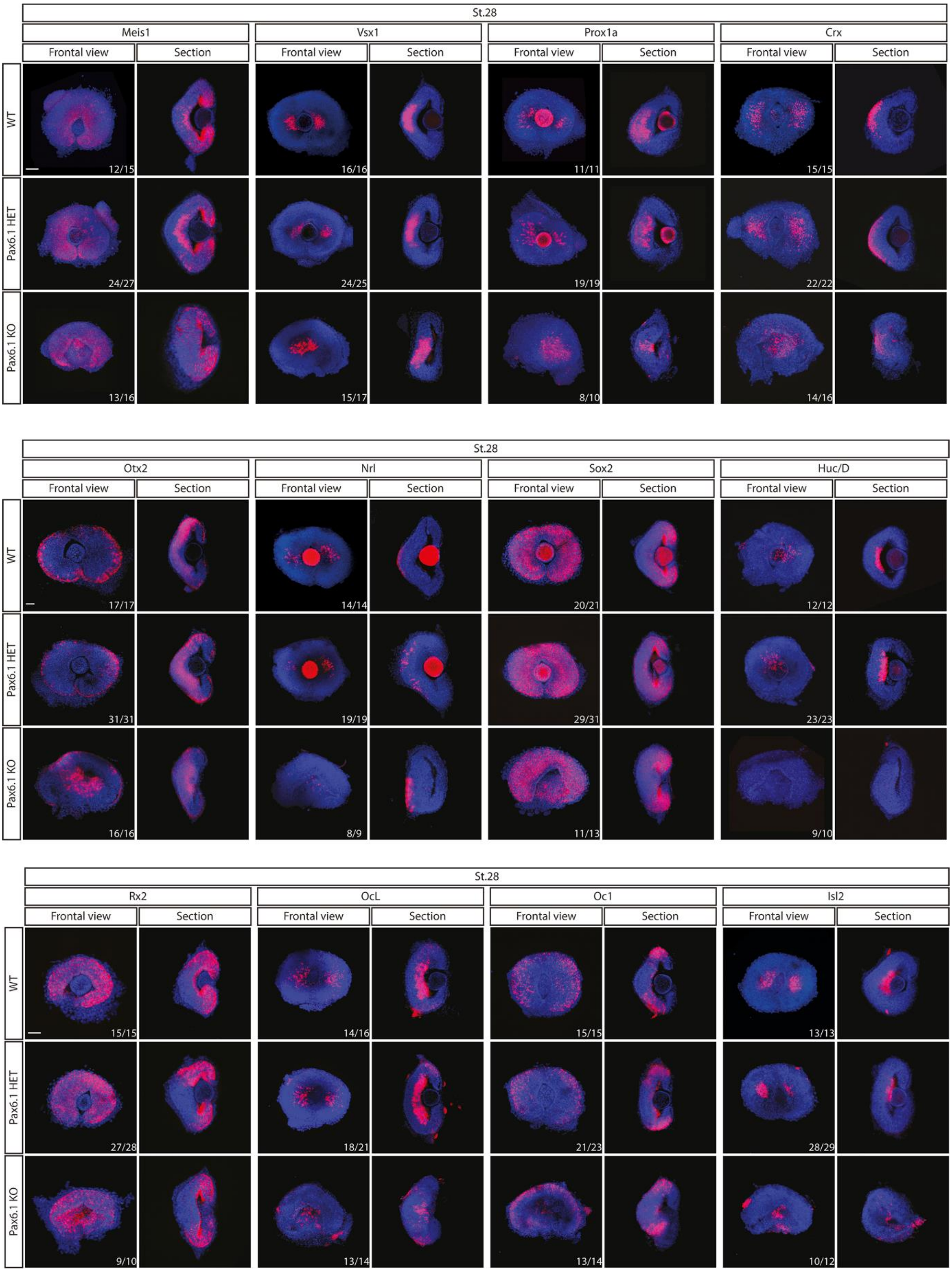

Supplementary Figure 7. Comparison of the expression patterns of selected genes (*Rx2, Sox2, HuC/D*, *Isl2*, *Oc1*, *OcL*, *Meis1*, *Vsx1*, *Prox1, Otx2, Nrl, Crx*) in the retina between WT, *Pax6.1* heterozygote and homozygote at the stage 28. Expression of examined genes remained unchanged in *Pax6.1* homozygote mutant. Scale bar: 50µm

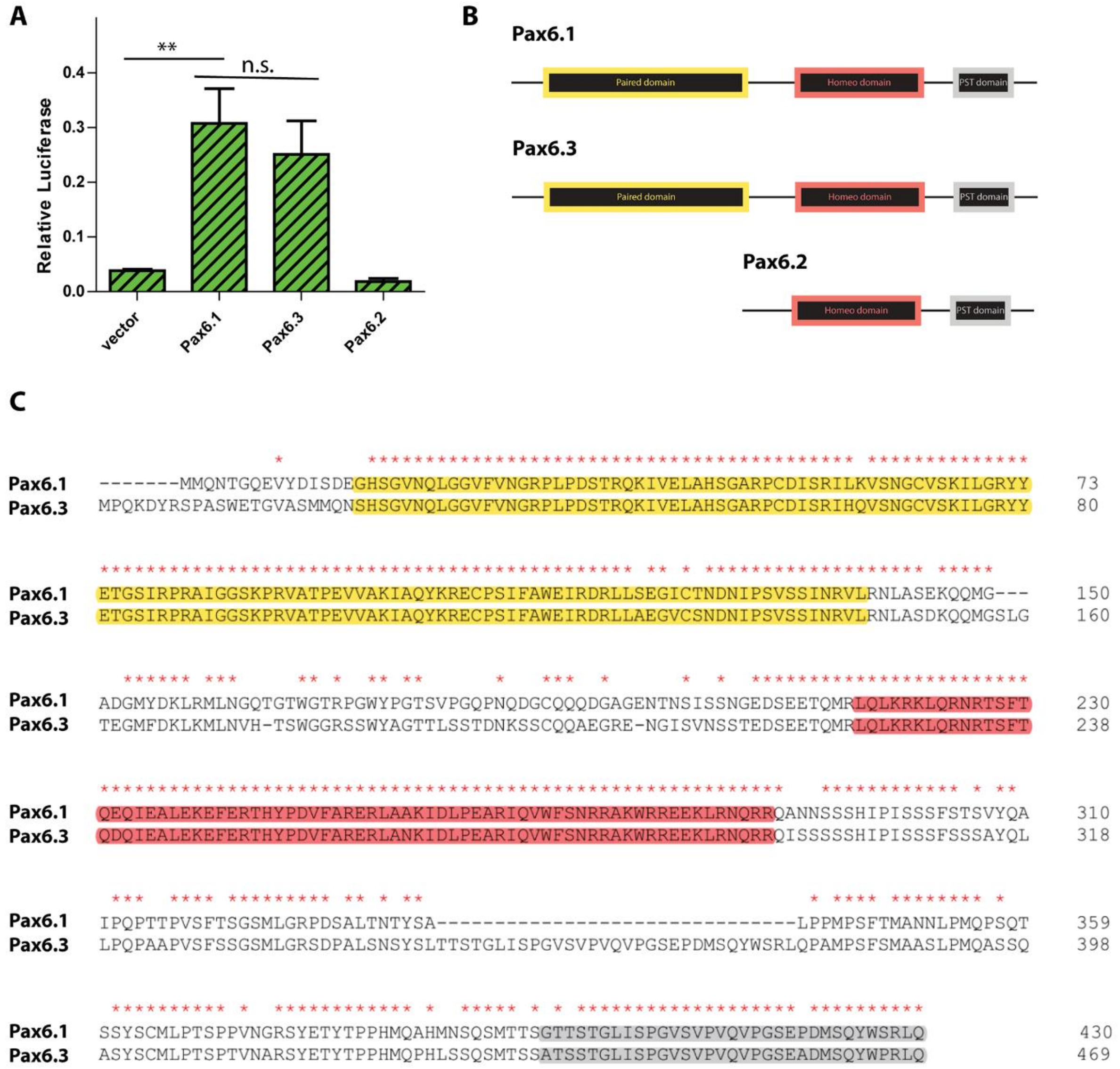

Supplementary Figure 8. Pax6.1 and Pax6.3 proteins share structural and functional properties. **(A)** Transient transfection assay shows comparable transactivation properties of Pax6.1 and Pax6.3 on Pax-responsive luciferase reporter gene. **(B)** Schematic diagram of domain structure of Pax6.1, Pax6.2, and Pax6.3. **(C)** Amino acid sequence alignment of medaka Pax6.1 and Pax6.3.

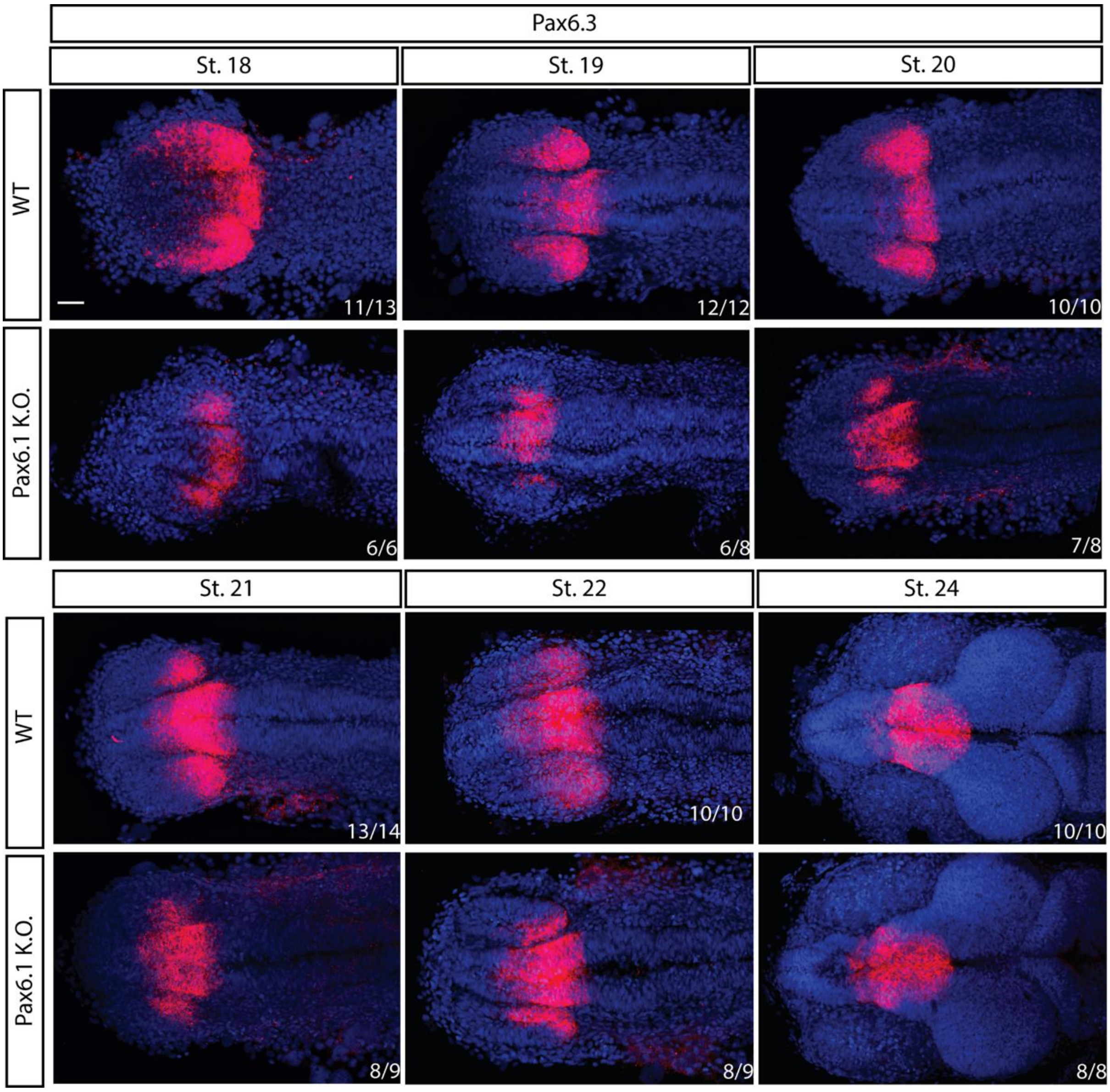

Supplementary Figure 9. *Pax6.3* expression pattern analysis in the *Pax6.1* mutant. The expression domain of *Pax6.3* in the eye is not enhanced but rather reduced in *Pax6.1* mutant.

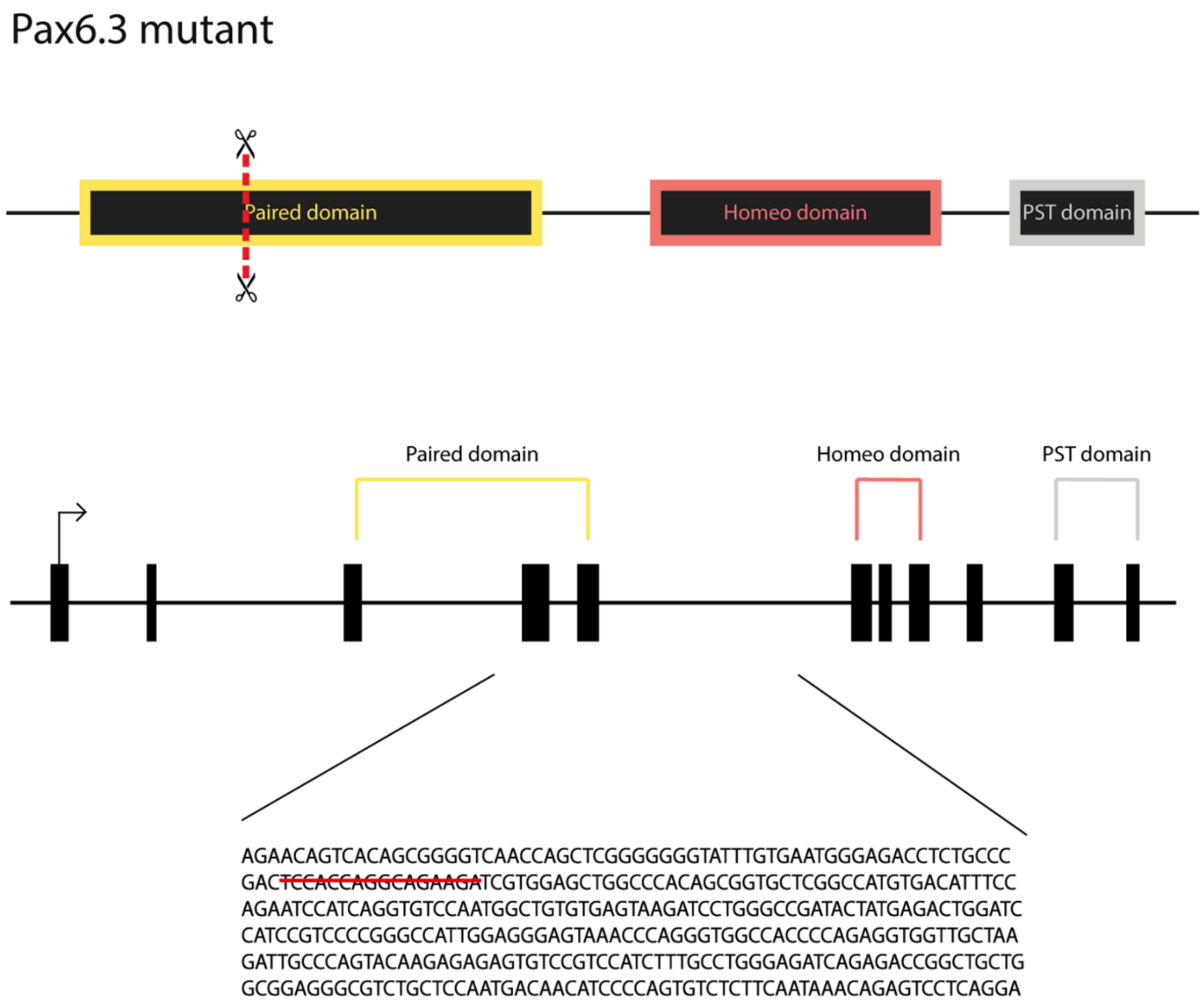

Supplementary Figure 10. Schematic representation of mutation in *Pax6.3* mutant. Deletion of 16 bp generated by genome editing leads to changes in amino acid structure of the protein and the appearance of the early stop codon.

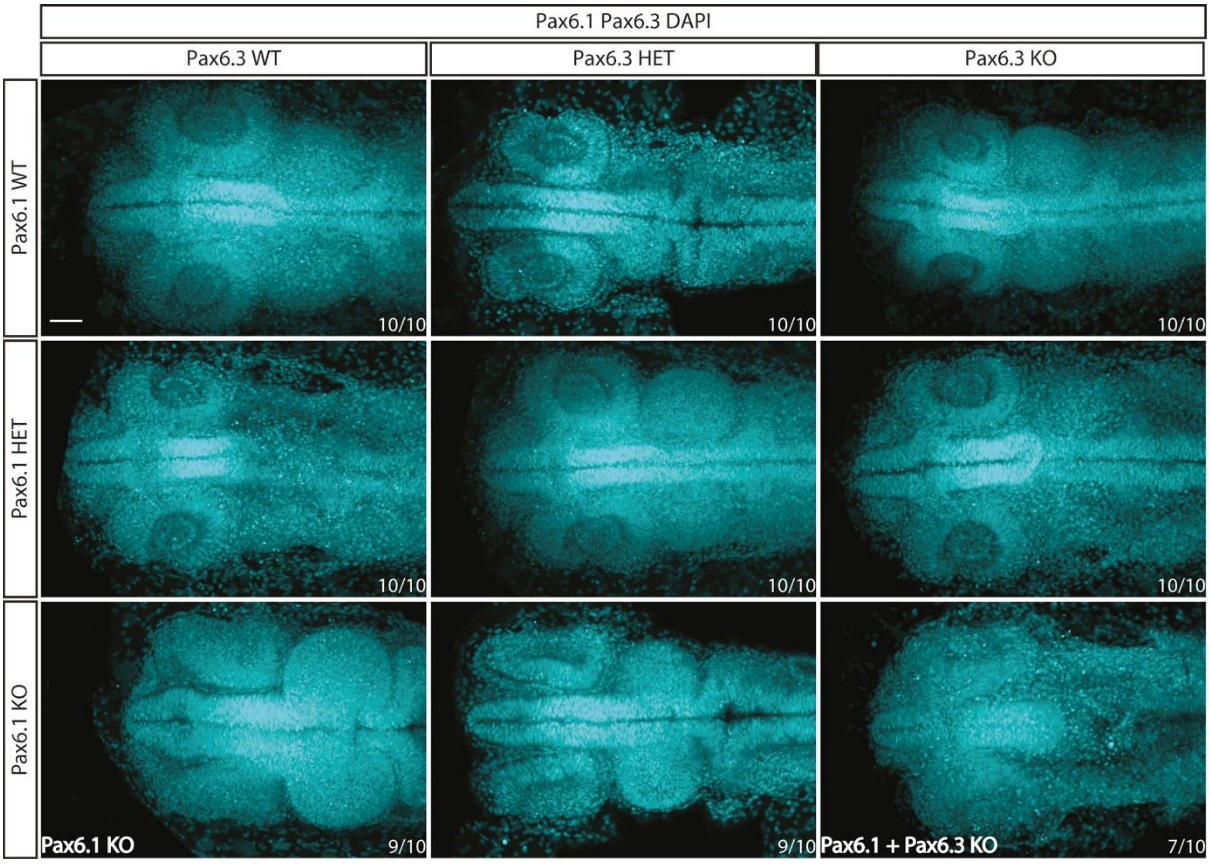

Supplementary Figure 11. The morphological analysis of *Pax6.1* and *Pax6.3* single mutants and *Pax6.1/Pax6.3* double mutant at stage 23 using DAPI staining. The retina of *Pax6.1* mutant and *Pax6.1/Pax6.3* double mutant is malformed and lenses are not present. On the other hand, no abnormalities were detected in *Pax6.3* mutant. Scale bar: 50µm

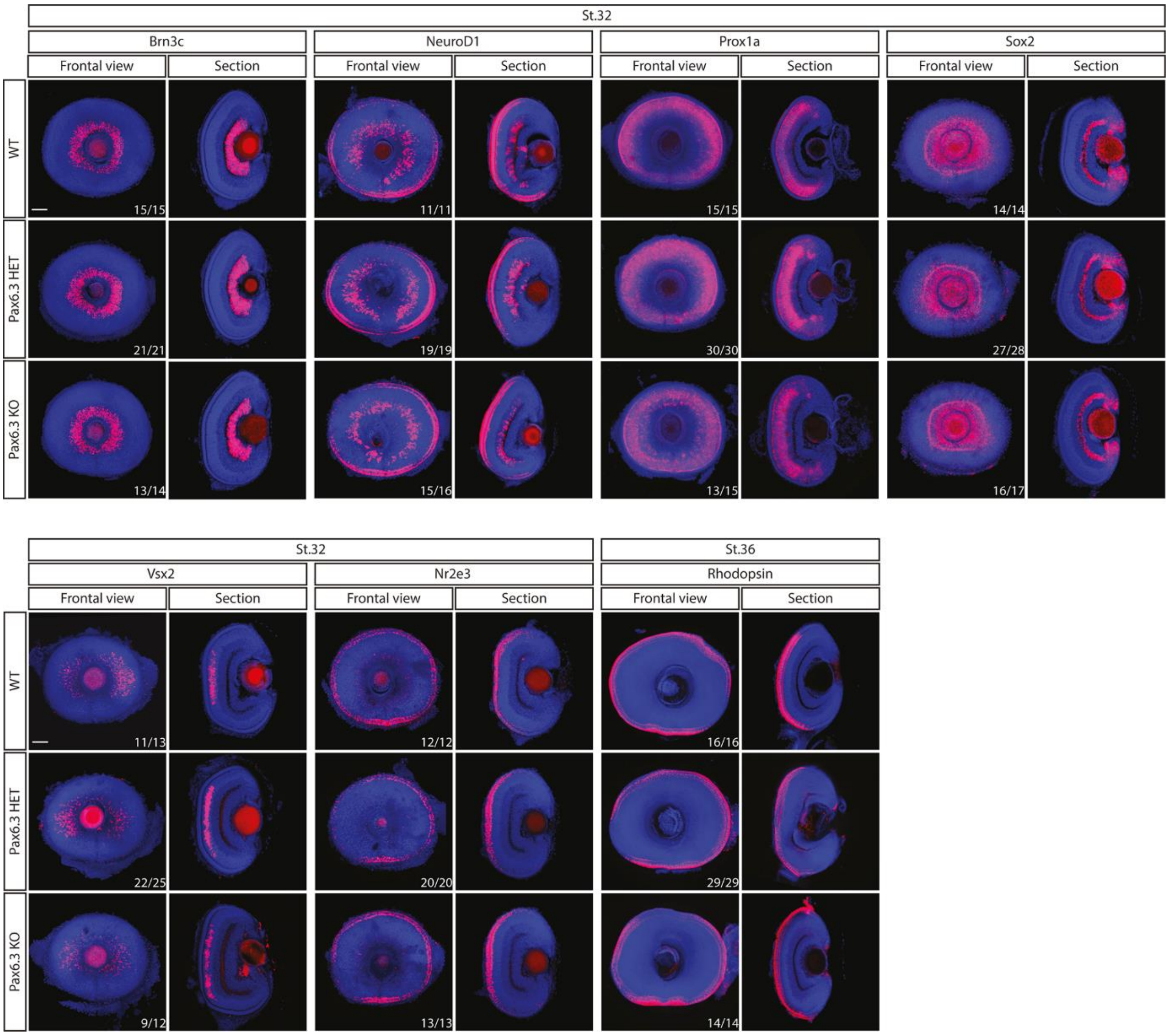

Supplementary Figure 12. Comparison of the *Brn3c*, *NeuroD1*, *Sox2*, *Vsx2*, *Prox1*, *Nr2e3* and *Rhodopsin* expression between wildtype, *Pax6.3* heterozygote and homozygote. All analyzed genes are expressed in the corresponding area indicating presence of all retinal cell types in *Pax6.3* mutant. Scale bar: 50µm

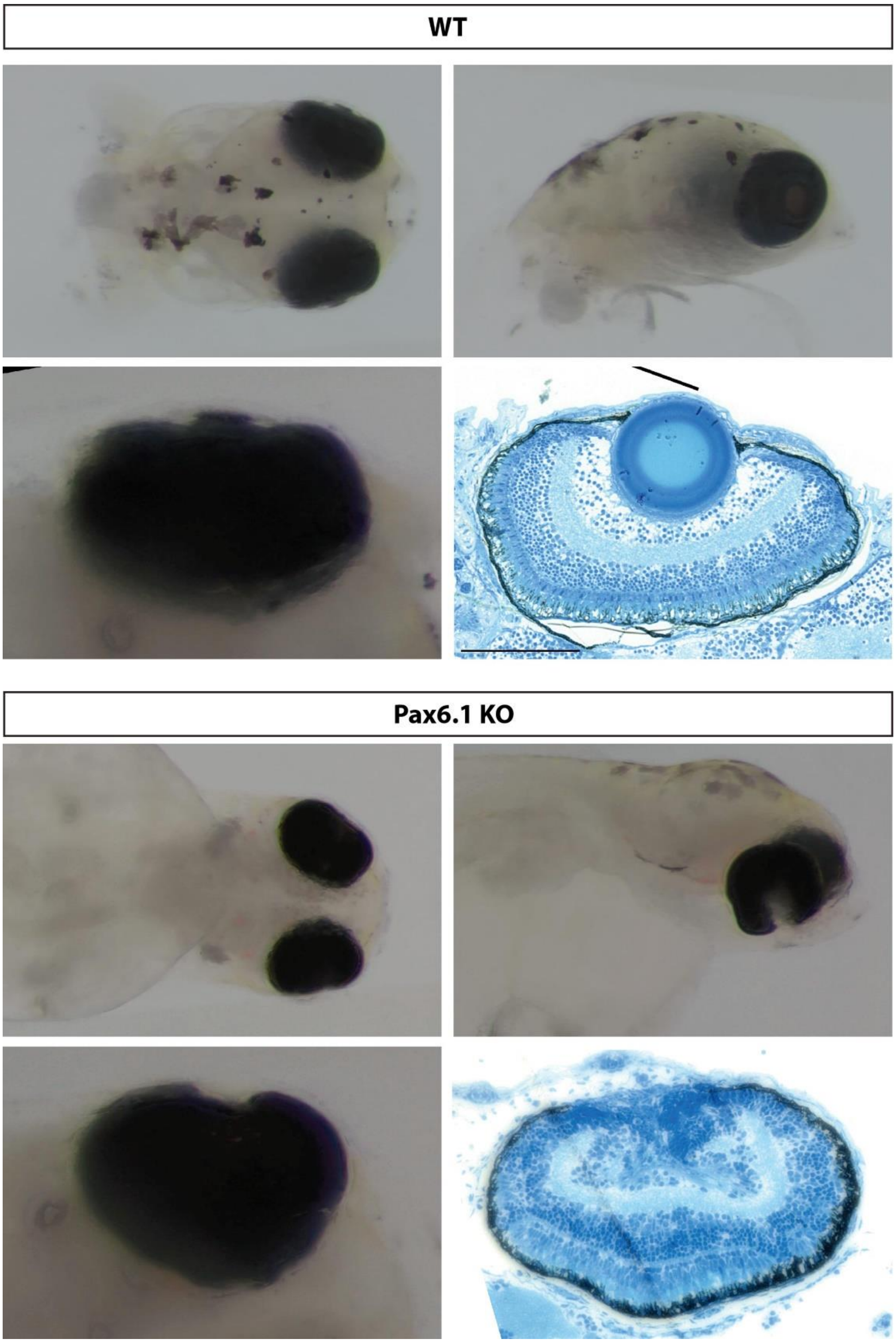

Supplementary Figure 13. A comparison of wt and Pax6.1 mutant homozygote fish at 7 days post fertilization. Top panels show dorsal and lateral views, respectively. Bottom panels show lateral views of eye and crossections, respectively. The lack of lens in mutants causes a structural collapse of the anterior chamber.
